# Turbulence in breaking mountain waves and atmospheric rotors estimated from airborne *in situ* and Doppler radar measurements

**DOI:** 10.1002/qj.2604

**Published:** 2015-09-24

**Authors:** Lukas Strauss, Stefano Serafin, Samuel Haimov, Vanda Grubišić

**Affiliations:** ^1^ Department of Meteorology and GeophysicsUniversity of ViennaAustria; ^2^ Department of Atmospheric ScienceUniversity of WyomingLaramieWYUSA; ^3^ Earth Observing LaboratoryNational Center for Atmospheric ResearchBoulderCOUSA

**Keywords:** mountain waves, wave breaking, boundary‐layer separation, rotors, airborne Doppler radar, energy dissipation rate, aviation turbulence

## Abstract

Atmospheric turbulence generated in flow over mountainous terrain is studied using airborne ***in situ*** and cloud radar measurements over the Medicine Bow Mountains in southeast Wyoming, USA. During the NASA Orographic Clouds Experiment (NASA06) in 2006, two complex mountain flow cases were documented by the University of Wyoming King Air research aircraft carrying the Wyoming Cloud Radar.

The structure of turbulence and its intensity across the mountain range are described using the variance of vertical velocity $\sigma_{w}^{2}$ and the cube root of the energy dissipation rate *ɛ* ^1/3^ (EDR). For a quantitative analysis of turbulence from the cloud radar, the uncertainties in the Doppler wind retrieval have to be taken into account, such as the variance of hydrometeor fall speed and the contamination of vertical Doppler velocity by the horizontal wind. A thorough analysis of the uncertainties shows that 25% accuracy or better can be achieved in regions of moderate to severe turbulence in the lee of the mountains, while only qualitative estimates of turbulence intensity can be obtained outside the most turbulent regions.

Two NASA06 events exhibiting large‐amplitude mountain waves, mid‐tropospheric wave breaking, and rotor circulations are examined. Moderate turbulence is found in a wave‐breaking region with $\sigma_{w}^{2}$ and EDR reaching 4.8 m^2^ s^−2^ and 0.25 m^2/3^ s^−1^, respectively. Severe turbulence is measured within the rotor circulations with $\sigma_{w}^{2}$ and EDR respectively in the ranges of 7.8–16.4 m^2^ s^−2^ and 0.50–0.77 m^2/3^ s^−1^. A unique result of this study is the quantitative estimation of the intensity of turbulence and its spatial distribution in the interior of atmospheric rotors, provided by the radar‐derived turbulence fields.

## 1 Introduction

Atmospheric flow over mountainous terrain can give rise to a variety of turbulent atmospheric processes. Sailplane (glider) pilots, soaring along mountain waves to reach higher altitudes, were among the first to recognize that the air in the vicinity of a mountain range is likely to bear considerable turbulence (Hirth, 1933). Early pilot reports of severe turbulence encounters at low altitudes soon sparked the interest of atmospheric scientists (Kuettner, 1938). Since the 1950s, various observational efforts have been undertaken to further the understanding of mountain waves and associated turbulent phenomena. During the Sierra Wave Project (Sierra Nevada, California, 1951–55), Holmboe and Klieforth (1957) and Kuettner (1959) identified low‐level circulation regions (‘atmospheric rotors’) in the lee of the Sierra Nevada, as the origin of severe turbulence below large‐amplitude lee waves.

However, mountain‐induced turbulence is not limited to the lowest layers of the troposphere. When mountain waves propagate upward, they can steepen due to decreasing air density or as they approach a critical level, start to overturn, and eventually break, leading to vigorous turbulent mixing (Dörnbrack, 1998; Sharman *et al.* 2012). Wave breaking at the tropopause or in the lower stratosphere has been observed occasionally by research aircraft and ground‐based remote sensors (e.g. Lilly and Kennedy, 1973; Lilly and Lester, 1974; Ralph *et al.*, 1997; Doyle *et al.*, 2005). In contrast, to the best of our knowledge, only four studies documenting aircraft encounters of mid‐tropospheric wave breaking exist to date (Lilly, 1978; Smith, 1987; Jiang and Doyle, 2004; Elvidge *et al.* 2014), including observations from the Alpine Experiment (ALPEX, 2015) and the Mesoscale Alpine Programme (MAP; Bougeault *et al.*, 2001).

In many of the above studies, research aircraft were key in characterizing atmospheric turbulence over mountainous terrain. However, use of aircraft near mountains frequently represents a delicate trade‐off between flying through the regions of scientific interest and ensuring safety of crew and equipment aboard. The actual hazard posed by mountain‐induced turbulence has been underlined by several investigations of severe turbulence encounters (e.g. Doyle *et al.*, 2005; Ólafsson and Ágústsson, 2009; Ágústsson and Ólafsson, 2014) and by reports of aviation incidents, e.g. the forced emergency landing of a DC‐8 cargo jet during the 1992 Colorado Front Range windstorm (Carney *et al.* 1995; Clark *et al.* 2000).

In recent years, the advancement of modern remote‐sensing instruments, and the possibility of operating them aboard aircraft, has offered new measurement approaches for the study of mountain airflows (Banta *et al.* 2013). By the nature of their measuring principle, these instruments lend themselves to the study of atmospheric turbulence from afar, thereby avoiding the need of flying directly into the turbulent regions of interest.

In this study, we make use of measurements by the Wyoming Cloud Radar (WCR), a W‐band Doppler and polarimetric pulsed radar, carried aboard the University of Wyoming King Air (UWKA) research aircraft. Previous deployments of WCR include the Dynamics and Chemistry of Marine Stratocumulus Experiment (DYCOMS‐II; Stevens *et al.*, 2003) and the Terrain‐induced Rotor Experiment (T‐REX; Grubišić *et al.*, 2008). T‐REX (Sierra Nevada, California, 2006) was the most recent major effort organized to investigate aspects of mountain‐induced turbulence related to mountain waves, rotors, and boundary‐layer dynamics. Unfortunately, only limited additional insight into these phenomena could be gained from WCR measurements in T‐REX due to the lack of moisture in Owens Valley and consequent unsatisfactory radar backscatter.

Prior to T‐REX, in January and February 2006, the NASA Winter Orographic Clouds Experiment (NASA06) was conducted over the Medicine Bow Mountains (MBM) in southeastern Wyoming, deploying UWKA and WCR in a moist midlatitude wintertime environment. Recently, French *et al.* (2015) and Grubišić *et al.* (2015)* provided a detailed analysis of two NASA06 events exhibiting large‐amplitude mountain waves. In their studies, they revealed the presence of large atmospheric rotors and the key role of mid‐tropospheric gravity‐wave breaking in steering the flow dynamics on both days. In the present work, we re‐examine the observational data set collected during the two wave events and extend previous analyses by the estimation of the intensity and spatial distribution of turbulence in mountain‐wave‐induced turbulent processes.

WCR measurements have previously been used for quantitative estimation of turbulence parameters; however, the application was limited to the marine boundary layer (Lothon *et al.* 2005). There, vertical profiles of the variance of vertical air velocity and energy dissipation rate were in good agreement with complementary aircraft *in situ* measurements. In our study, the inherent inhomogeneity of the flow field over mountainous terrain and the changes in attitude of the aircraft when encountering turbulence at flight level pose significant challenges for the data analysis, which we will address in detail.

The goal of this article is twofold. First, to show that Doppler velocities from airborne single‐Doppler radar can effectively be used to detect locations of strong turbulence across mountain ranges and to obtain quantitative measures of turbulence, including upper bounds of the measurement uncertainty. Second, to apply this newly devised technique to the NASA06 data set, covering several complex mountain flow cases involving a rich variety of mountain‐induced turbulent processes.

The rest of this article is organized as follows. In section 2, we give a brief overview of the NASA06 campaign and the relevant characteristics of the aircraft instrumentation and radar. In section 3, we describe the parameters we use to quantify turbulence and provide a detailed analysis of their uncertainties. Section 4 contains results from three days of the NASA06 campaign. In section 5, we discuss the observed turbulent phenomena. Conclusions are drawn in section 6.

## 2 Field campaign and airborne instruments

FHO15 provide an extensive overview of the NASA06 experiment, including the topographic setting, airborne instruments, and their features and limitations. Geerts *et al.* (2011) also discuss data from NASA06 and provide details of the design of this and similar experiments over the MBM from 2006 to 2009. In this section, we summarize the observed cases and discuss the characteristics of the airborne instrumentation relevant for our study.

### 2.1 *Field campaign*

**Figure 1:**
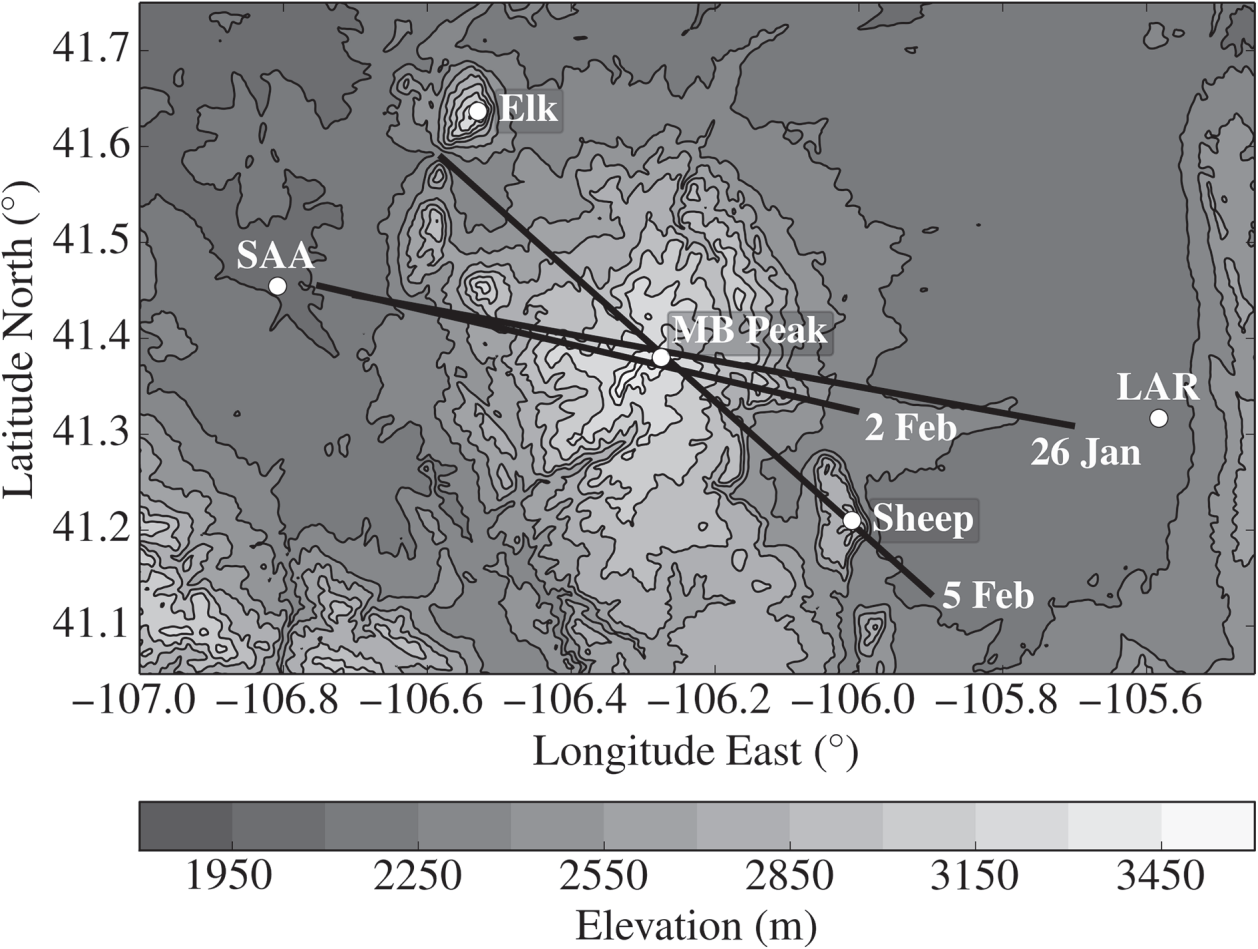
Topographic map of the Medicine Bow Mountains, southeastern Wyoming. White circles mark the location of Elk Mountain, Medicine Bow (MB) Peak (3662 m amsl), Sheep Mountain, Shively Field Airport in Saratoga (SAA) and Laramie Regional Airport (LAR). Black solid lines indicate the orientation of research flights on 26 January, 2 February, and 5 February 2006.

The NASA06 experiment took place over the MBM in southeastern Wyoming in January and February 2006. Figure 1 gives an overview of the MBM topography and a sketch of the orientation of research flights on the interesting days of the campaign.

During NASA06, UWKA flights consisted of series of straight‐and‐level legs crossing the highest elevations of the MBM approximately in and against the mean wind direction. Data from three of these flights is analysed here.

By design, the NASA06 experiment focused primarily on the fine structure of deep wintertime orographic clouds and the role of aerosols in the formation of orographic precipitation (Geerts *et al.* 2011). However, on two days of the campaign, 26 January and 5 February 2006, stable upstream conditions and favourable mesoscale dynamic forcing led to enhanced atmospheric response to the underlying topography, including large‐amplitude lee waves, gravity‐wave breaking, and strong low‐level turbulence. In addition to the two wave events, a third day, 2 February 2006, is included in the analysis as a reference case for non‐wave‐induced boundary‐layer turbulence.

### 2.2 *Measurement platform and instruments*

A comprehensive characterization of the aircraft *in situ* instrumentation and the cloud radar is provided in FHO15 and, more generally, in Wang *et al.* (2012) and UWKA (2015), and in Damiani and Haimov (2006) and WCR (2015).

#### 2.2.1 *UWKA research aircraft*

The UWKA research aircraft is a specially instrumented Beechcraft Super King Air 200T. For the estimation of turbulence parameters, we make use of its high‐rate (25 Hz) measurements of the three components of the wind, obtained from a five‐hole ‘gust’ probe, located on an extended nose boom, and measurements of air temperature and static pressure (Brown *et al.* 1983). The precision of the wind measurement is approx. ±0.1 m s^−1^ for the horizontal along‐track and cross‐track wind components *u* and *v*, and ±0.05 m s^−1^ for the vertical wind component *w* (FHO15).

The second relevant instrument aboard UWKA is the Universal Indicated Turbulence System (UITS), commonly referred to as ‘MacCready Turbulence Meter’. The UITS design is based on a method proposed by MacCready (1962, 1964) for the determination of the rate of dissipation of turbulent kinetic energy. On UWKA, the MacCready Turbulence Meter is used primarily as a real‐time, on‐flight indicator of turbulence (Feng, 2001). In this study, data from the instrument is used for comparison with dissipation rates obtained from spectral analysis of high‐rate wind data.

#### 2.2.2 *Wyoming Cloud Radar (WCR)*

The Wyoming Cloud Radar (WCR) is a 95 GHz pulsed, fixed multi‐antenna Doppler radar, designed to provide high‐resolution data of the structure and dynamics of clouds. During NASA06, WCR was operated in three‐antenna mode, with beams pointing to the zenith, nadir and 30° down‐forward directions when the aircraft flew straight and level. The dwell time of the radar (the time it takes to collect one radar profile) was approximately 32 ms, resulting in an along‐track sampling distance of approx. 3 m at an average true airspeed of approx. 100 m s^−1^. The radar resolution in the vertical (distance between range gates) was 30 m and the radar pulse volume at 1 km had a size of approx. 10 × 10 × 30 m^3^. Radar backscattered reflectivity during NASA06 was predominantly from homogeneous ice clouds, consisting mostly of spherically shaped ice particle aggregates (FHO15). In the analysis of Doppler velocities, contamination by ground clutter was avoided by using radar data starting at 100 m above ground level.

FHO15 combined the signals from the radar nadir and down‐fore beam for dual‐Doppler analysis in order to determine the flow across the MBM in the along‐track measurement plane. However, in the present work, only data from the nadir and zenith beams is used and it is shown that quantitative estimates of turbulence intensity can be obtained from single‐Doppler measurements alone.

## 3 Quantitative turbulence estimates from airborne ***in situ*** and Doppler radar measurements

*In situ* measurements from UWKA have previously been used to study boundary‐layer turbulence in mountainous environments (Darby and Poulos, 2006; Jiang *et al.* 2010; Geerts *et al.* 2011). In the present work, we extend these studies, determining the intensity and spatial distribution of turbulence in mountain‐induced phenomena from both UWKA and WCR data.

Quantitative estimation of turbulence requires the selection of suitable turbulence metrics. For the data at hand, the turbulent kinetic energy TKE, the variance of vertical velocity $\sigma_{w}^{2}$, and the rate of dissipation of turbulent kinetic energy to the power of one third *ɛ* ^1/3^, or EDR, have emerged as appropriate turbulence parameters.

### 3.1 *Turbulence analysis approach*

The mountain airflow cases under consideration are characterized by highly inhomogeneous turbulent fields forced from aloft by the presence of gravity waves. Thus, in contrast to more uniform boundary‐layer flows studied by Geerts *et al.* (2011) and Lothon *et al.* (2005), the assumption of horizontal homogeneity cannot be made here.

Wavelet transforms would lend themselves naturally to the analysis of inhomogeneous mountain flows, since they allow the structure of waves and turbulence to be resolved simultaneously in both spatial and wavenumber domains (Torrence and Compo, 1998). However, despite recent developments (e.g. Terradellas *et al.*, 2005; Woods and Smith, 2010), a well‐established method for quantitatively determining turbulence parameters, such as TKE, from wavelets does not exist to date.

In their review of the experimental investigation of atmospheric boundary‐layer turbulence, Druilhet and Durand (1997) commented on the issue of inhomogeneous data stating that ‘no general method exists for analysing inhomogeneous time series’ but that a ‘sample can be divided into more homogeneous sub‐samples and thus analysed by conventional techniques such as short Fourier transform’. We thus deal with the inherent inhomogeneity of the flow field by subdividing UWKA and WCR data into short segments, each of which is subjected to turbulence analysis individually.

The choice of the length of these segments must be made with care. Choosing the right segment length represents a trade‐off between obtaining statistically meaningful estimates of turbulence parameters and maintaining the homogeneity of turbulence within each data segment. This choice is tightly linked to the question of what scale separates the turbulent and mean parts of the flow. Once a segment length has been chosen, atmospheric motion only at smaller scales can be resolved.

**Figure 2:**
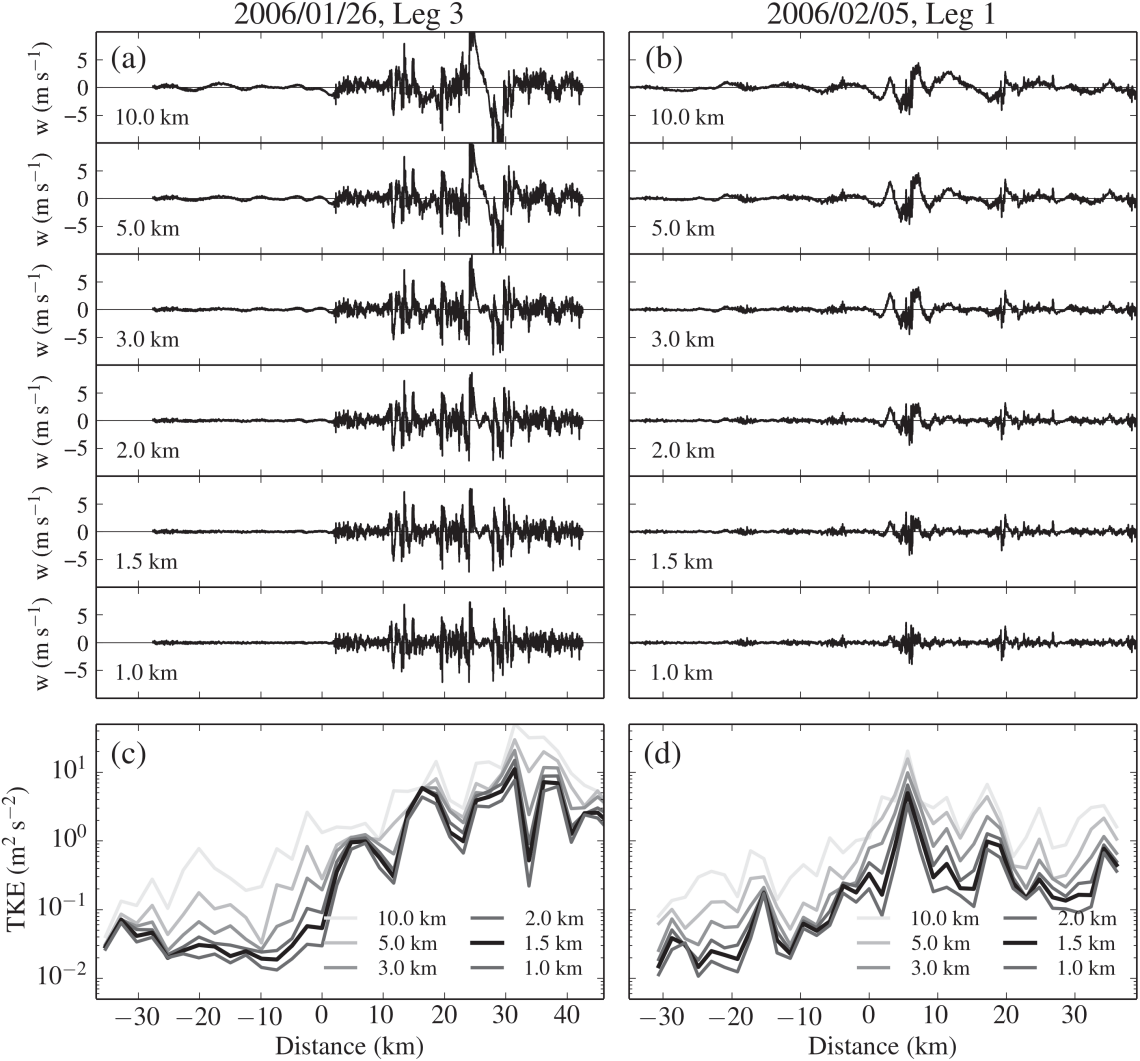
The effect of high‐pass filtering, using an equally weighted moving average with different window widths. Vertical velocity *w* for (a) Leg 3 on 26 January and (b) Leg 1 on 5 February 2006, for filter scales ranging from 10 to 1 km. (c,d) Turbulent kinetic energy TKE for the same flight legs and filter scales. TKE at filter scale 1.5 km, used throughout this work, is indicated in black. In this and later figures, distance is upwind (<0) or downwind (>0) of the MBM top. The times and altitudes of all flight legs are summarized in Table 1.

Figure 2(a,b) illustrates how the spatial series of the vertical wind component *w* change upon application of a high‐pass filter (subtraction of the equally weighted moving average from the raw signal) with decreasing filter scale. Obvious features of the mesoscale flow (e.g. upstream waves and lee‐side up‐ and downdraughts) are apparent from all filtered *w* series except for scales 1.5 and 1 km. Figure 2(c,d) reveals the effect of the high‐pass filtering on TKE, which we compute as half of the sum of variances of the filtered wind components $(\sigma_{u}^{2}+\sigma_{v}^{2}+\sigma_{w}^{2})/2$ along the leg. At and below a scale of 1.5 km, the largest portion of the variance of the signal due to mesoscale motions has been removed.

This test serves as a guideline for the choice of the length of the segments that are cut from the spatial series. For subsequent turbulence analysis, we proceed with a length of 1.5 km.

### 3.2 *Variance of vertical velocity*

Estimates of the variance of the horizontal along‐track (*u*) and cross‐track (*v*) wind components (for UWKA) and the vertical wind component (*w*, for both UWKA and WCR) are obtained from the linearly detrended wind data for each 1.5 km segment. For WCR, data from each radar range gate is used as individual spatial series.

Any complete measurement of a physical quantity requires the specification of the measurement uncertainty. Prior to this study, it was not clear whether the inherent inaccuracies in the measured wind velocities from airborne Doppler radar permit quantitative estimation of turbulence parameters in a spatially inhomogeneous airflow and in conditions of moderate flight‐level turbulence. In the following, we thoroughly consider all possible uncertainties in the computed variances.

**Figure 3:**
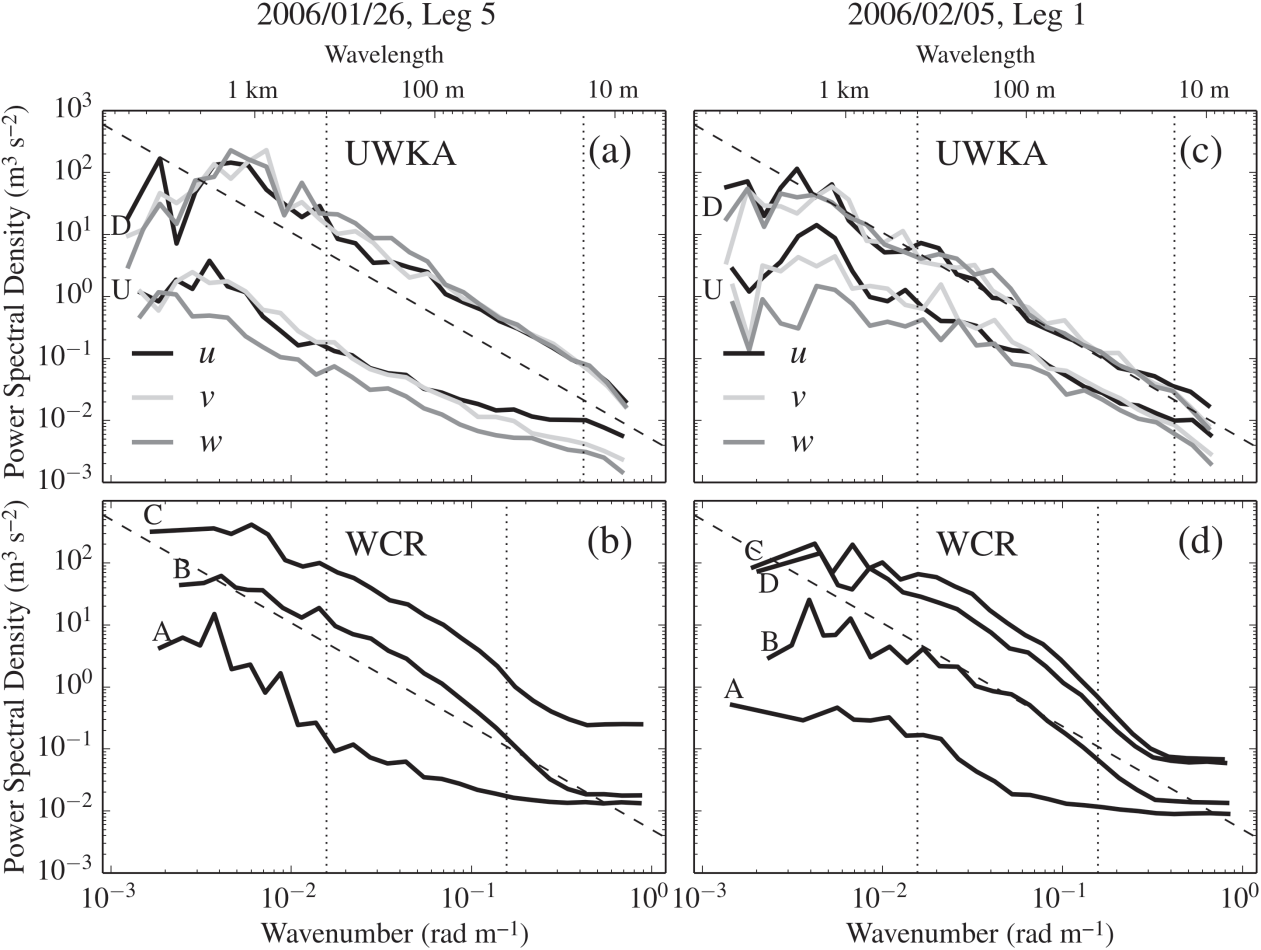
Example Fourier spectra computed from *in situ* wind components and radar Doppler velocity, measured along (a,b) Leg 5 on 26 January and (c,d) Leg 1 on 5 February in different regions of the flow. Panels (a) and (c) show averaged spectra from flight‐level data from stretches upstream (U) and downstream (D) of Medicine Bow Peak. Panels (b) and (d) contain averaged spectra from WCR data from regions A, B, C marked in Figure 9(b) and regions A, B, C, D marked in Figure 10(b). Raw spectra have been spectrally averaged using ten equal‐log‐interval bins per decade (*Δ*(*log*_10_ *k*) = 0.1). Vertical dotted lines indicate the range of wavenumbers *k* from which the energy dissipation rate is extracted (section 3.3 gives more information). Dashed solid lines mark the −5/3 spectral slope, expected for the inertial subrange. Section 3.2 gives a discussion of spectrum shapes.

As a first step, we inspect the power spectra of measured quantities for their noise level. Figure 3(a,c) refer to UWKA wind measurements taken in the relatively quiescent region upstream of the mountain and in the strongly turbulent region downstream of it. Spectra from the downstream region follow a −5/3 power law (indicative of the inertial subrange) from scales of roughly 400 m down to 15 m, while those from the upstream region display a flattening at the high‐wavenumber end, commonly associated with instrumental white noise. Similarly, Figure 3(b,d) refer to WCR vertical Doppler velocity from different regions of the flow. Most WCR spectra, from both up‐ and downstream of the mountain, exhibit an approximate −5/3 power law behaviour at scales between 400 and 40 m, while they only contain white noise at wavelengths shorter than 20 m. The level of uncorrelated white noise is highly variable, which is attributed to the Doppler radar system signal‐to‐noise (SNR) ratio.

In order to correct the measured variances for the contribution of noise to them, the level of white noise $\sigma_{\text{noise}}^{2}$ is extracted from the power spectra in each series segment. $\sigma_{\text{noise}}^{2}$ is then subtracted from the measured variance $\sigma_{w,\text{meas}}^{2}$.

Beyond the noise contributions, which are readily determined from power spectra, there are a number of uncertainties pertaining to the moving measurement platform and to characteristics of the remote‐sensing instrument: (i) the uncertainty in the determination of aircraft motion and attitude; (ii) the limited accuracy of the beam pointing‐angle calibration; (iii) the variance of hydrometeor fall speed; (iv) the radar pulse‐volume‐averaging (PVA) effect; and (v) the contamination of radial Doppler velocity *v*_r_ by the horizontal wind.

The error in the determination of aircraft motion and attitude (Haimov and Rodi, 2013) contributes an uncertainty of $\sigma_{\text{ac}}^{2}≃0.01m^{2}s^{-2}$ to the measured vertical wind velocity. Calibration of the radar‐beam pointing angle is needed to correctly remove the aircraft motion from measured Doppler velocities. For NASA06, a realistic estimate of its error and the consequent uncertainty is $\sigma_{\text{ba}}^{2}≲0.09m^{2}s^{-2}$, based on considerations by Haimov and Rodi (2013). The variance of hydrometeor fall speed contributes to the measured variance $\sigma_{v_{r}}^{2},\text{meas}$. FHO15 obtained an estimate of the fall velocity *v*_t_ of ice particle aggregates of 1 m s^−1^ and standard deviation of 0.2 m s^−1^, leading to a maximum positive bias in the radar‐measured variance of $\sigma_{v_{t}}^{2}≃0.04m^{2}s^{-2}$.

The averaging effect of the finite radar pulse volume (Srivastava and Atlas, 1974; Lothon *et al.* 2005) is responsible for the bending‐down of spectral energy density at the higher wave numbers for WCR spectra (Figure 3(b,d)). Lothon *et al.* (2005) corrected for this effect assuming a von Kármán energy spectrum and measuring the integral scale of turbulence *L*. In the present study, reliable estimates of *L* cannot be obtained from short data segments. We thus compensate for the PVA effect, which implies a loss of variance $\sigma_{\text{PVA}}^{2}$, assuming that it depends linearly on the along‐track (longitudinal) pulse‐averaging filter width. The latter is in turn proportional to the distance from the radar. In absence of a suitable estimate of *L*, $\sigma_{\text{PVA}}^{2}$, added to the measured variance in each leg segment, is only a rough correction and we set a conservative estimate for the error of this correction to $\sigma_{\text{PVA},\text{err}}^{2}≃\pm 0.25\sigma_{\text{PVA}}^{2}$.

Finally, cross‐contamination of velocity components occurred because a steady aircraft attitude could not always be maintained during NASA06 flights, in particular when UWKA encountered moderate, or stronger, turbulence at flight level. Under such conditions, the fixed up/down‐pointing radar beams deviated from zenith/nadir, leading to a contamination of the radial Doppler velocity *v*_r_ by the horizontal along‐track and cross‐track wind components. Far from flight level, however, these are unknown and can differ by as much as 30 and 10 m s^−1^, respectively. This adds an additional uncertainty in the estimation of $\sigma_{w}^{2}$. The maximum contribution of the horizontal wind contamination (HC) to $\sigma_{v_{r}}^{2},\text{meas}$ is highly variable along each flight leg and depends on the pitch and roll angle variation in each leg segment. As shown in Appendix 1, variances of pitch and roll along a leg segment tend to cause a positive bias in the measured variance ($+\sigma_{\text{HC},A}^{2}$), while non‐zero mean pitch and roll can lead to both an increase and decrease in the variance ($\pm \sigma_{\text{HC},B}^{2}$).

Summing up all of the above uncertainties, the variance of vertical velocity $\sigma_{w}^{2}$ is given by

(1) $$\sigma_{w}^{2}=\sigma_{w,\text{meas}}^{2}-\sigma_{\text{noise}}^{2}+\left\{\begin{array}{c} 0\\ -\sigma_{\text{ac}}^{2} \end{array}\right.$$

for UWKA measurements and by

(2) $$\begin{array}{lll} \sigma_{w}^{2}= & \sigma_{v_{r}}{,\text{meas}}^{2}-\sigma_{\text{noise}}^{2}+\sigma_{\text{PVA}}^{2}\; & \;\\ & \;+\;\left\{\;\;\;\;\begin{array}{ccc} +\sigma_{\text{PVA},\text{err}}^{2} & \;+\sigma_{\text{HC},B}^{2}\\ -\sigma_{\text{PVA},\text{err}}^{2} & -\sigma_{\text{HC},A}^{2} & \;-\sigma_{\text{HC},B}^{2}\;-\;\sigma_{\text{ac}}^{2}\;-\;\sigma_{\text{ba}}^{2}\;-\;\sigma_{v_{t}}^{2} \end{array}\right.\; & \end{array}$$

for WCR measurements, where the upper and lower lines in braces represent the upper and lower uncertainty bounds, respectively. For WCR, the relative uncertainty of $\sigma_{w}^{2}$ is defined as

(3) $$R=\frac{\sigma_{\text{PVA},\text{err}}^{2}+\sigma_{\text{HC},A}^{2}+\sigma_{\text{HC},B}^{2}+\sigma_{\text{ac}}^{2}+\sigma_{\text{ba}}^{2}+\sigma_{v_{t}}^{2}}{\sigma_{v_{r},\text{meas}}^{2}-\sigma_{\text{noise}}^{2}+\sigma_{\text{PVA}}^{2}},$$

where the numerator and denominator stand for the maximum uncertainty and the signal, respectively. In the following sections, quantitative statements on turbulence intensity will be made only in regions of the flow with *R* ≤ 25%.

### 3.3 *Energy dissipation rate*

The second turbulence metric that we use is the cube root of the rate of dissipation of the turbulent kinetic energy EDR. Beyond its use in the scientific community, EDR has recently been established as an aircraft‐independent objective measure for turbulence intensity (ICAO, 2007; Sharman *et al.* 2014). EDR is related to the standard deviation of the vertical acceleration $\sigma_{\ddot{z}}$ on aircraft (MacCready, 1964; Cornman *et al.* 1995) and thus provides the link to the subjective feel of turbulence experienced by aircraft passengers and crew. In civil aviation, EDR values are linked to ‘turbulence categories’ used in pilot reports. Following newly‐proposed guidelines for medium‐sized commercial aircraft by Sharman *et al.* (2014), we adopted EDR thresholds of 0.014, 0.050, 0.125, 0.220, 0.350, 0.500 m^2/3^ s^−1^, respectively, for turbulence categories ‘smooth‐light’, ‘light’, ‘light‐moderate’, ‘moderate’, ‘moderate‐severe’, and ‘severe’.

For the estimation of EDR, we make use of the inertial dissipation technique (IDT; Champagne, 1978; Piper and Lundquist, 2004; Večenaj *et al.*, 2012). The IDT is based on the Kolmogorov form of the turbulent energy spectrum in the inertial subrange (Kolmogorov: 1941a, 1941b) stating that, under conditions of local isotropy and large Reynolds numbers, a range of wave numbers exists for which

(4) $$S_{i}(k)=\alpha_{i}{ɛ}^{2/3}k^{-5/3},$$

where *S*_*i*_(*k*) is the spectral energy density of velocity component *u*_*i*_={*u*,*v*,*w*} and *α*_*i*_ is the Kolmogorov constant *α*_*i*_={0.53,0.707,0.707} (Oncley *et al.* 1996; Piper and Lundquist, 2004).

One of the advantages of using EDR as a turbulence indicator instead of $\sigma_{w}^{2}$ or TKE arises from the EDR being estimated from the energy at smaller turbulent scales, which are affected to a lesser degree by preliminary filtering or the choice of the segment length. A disadvantage of the method is the more stringent requirement of the presence of a ‘−5/3 region’ in the spectra and of local isotropy, limiting its applicability. For this work, we implemented the IDT as follows:

1. The raw wind data from each 1.5 km leg segment is linearly detrended and the power spectral density is computed using Welch's method (Welch, 1967).
2. Contrary to other studies (Oncley *et al.* 1996; Piper and Lundquist, 2004) we do not define a fixed wavenumber range from which EDR is extracted. Instead, for each spectrum, we determine the region of the spectrum with the best correspondence to the −5/3 slope, based on several least‐squares fits to the spectrum within the wavenumber limits that are indicated by the vertical lines in Figure 3. The input parameters to this procedure are: the wavenumber limits of the fit (2*π*/400 to 2*π*/15 m^−1^ for UWKA and 2*π*/400 to 2*π*/40 m^−1^ for WCR data), the minimum window width for the fit (*Δ*(*log*_10_ *k*) = 0.7), and the maximum allowed deviation from the −5/3 slope (10%). The freedom in determining the inertial subrange is needed in order to handle varying scales of turbulence, depending on the region of the mountain flow under consideration. It also helps to deal with the range‐dependent pulse‐volume‐averaging effect of the Doppler radar.
3. EDR_*i*_ is estimated from the spectrum of each wind component *u*_*i*_ as
  (5) $${\text{EDR}}_{i}={ɛ}_{i}^{1/3}={\left(\bar{\frac{S_{i}(k)k^{5/3}}{\alpha_{i}}}\right)}^{\;1/2},$$
  where the overline bar stands for the arithmetic mean taken over all wave numbers *k* within the detected inertial subrange.

**Figure 4:**
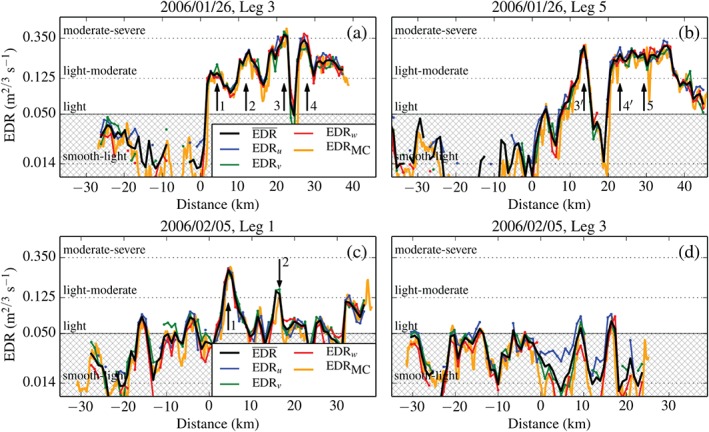
Aircraft *in situ* measurements of dissipation rate (*ɛ* ^1/3^, or EDR) along flight legs crossing the MBM on (a,b) 26 January and (c,d) 5 February. EDR_*u*_, EDR_*v*_, and EDR_*w*_ are the dissipation rate estimates from the three wind components and $\bar{\text{EDR}}$ is their mean. EDR_MC_ corresponds to dissipation rates from the MacCready Turbulence Meter, used for comparison. Vertical arrows mark the location of peaks in turbulence intensity, discussed in section 4. Arrows labelled with primed numbers refer to the same peak in turbulence intensity but at a later point in time. Hatched regions indicate values lower than 0.05 m^2/3^ s^−1^ which are not reliable (Figure 5).

Figure 4 includes the cross‐mountain variation of EDR at flight level along flight legs on 26 January and 5 February. Very good agreement can be seen between the EDR measurement by the MacCready Turbulence Meter (EDR_MC_) and the ‘log‐mean’ EDR estimate, which we define as

(6) $$\begin{array}{lll} \bar{\text{EDR}} & =10^{\left\{\mathrm{lo}g_{10}({\text{EDR}}_{u})+\mathrm{lo}g_{10}({\text{EDR}}_{v})+\mathrm{lo}g_{10}({\text{EDR}}_{w})\right\}/3}.\; & \end{array}$$

**Figure 5:**
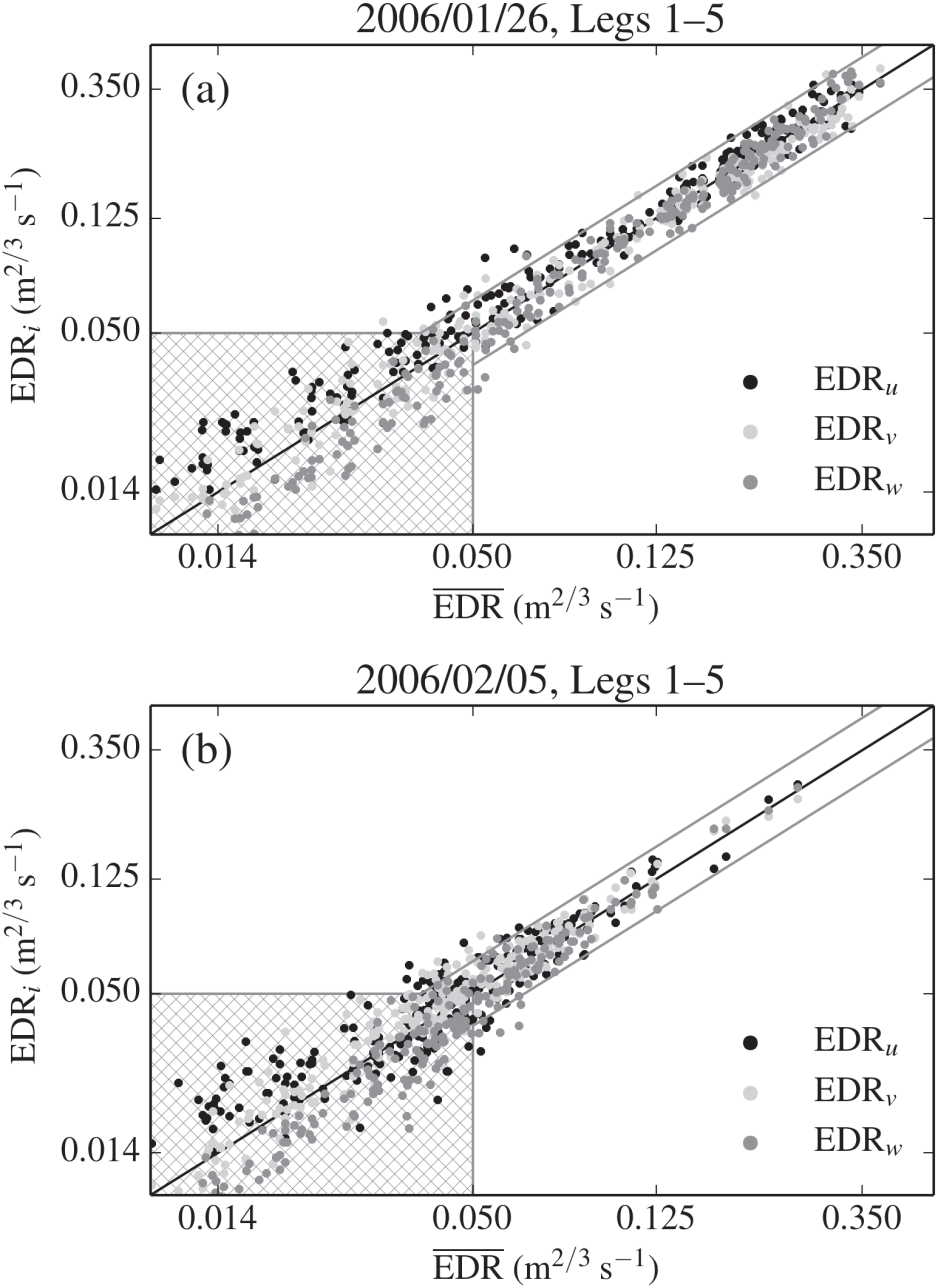
Scatter of individual estimates EDR_*u*_, EDR_*v*_, and EDR_*w*_ around their log‐mean $\bar{\text{EDR}}$ (black solid line) for all flight legs on (a) 26 January and (b) 5 February, respectively. The hatched regions indicate low EDR estimates (<0.05 m^2/3^ s^−1^), which are not reliable (see discussion in the text). The grey solid lines mark the 95% (±2*σ*) interval around $\bar{\text{EDR}}$, representative of the uncertainty of individual EDR estimates.

Considerable scattering of individual EDR estimates around $\bar{\text{EDR}}$ is also visible. This scatter is particularly pronounced in the region of relatively low turbulence upstream of the mountain top. There are several possible origins of this scatter: anisotropic turbulence, statistical scatter of EDR estimates (related to the limited extent of the leg segments), and uncertainty in the determination of the inertial subrange. Figure 5 contains two scatter diagrams consisting of EDR estimates from all legs flown on 26 January and 5 February, respectively. For values of $\bar{\text{EDR}}<0.05m^{2/3}s^{-1}$, corresponding to the hatched region, a clear tendency of the EDR estimate from the along‐track horizontal wind component (EDR_*u*_) to be larger than those from the vertical wind component (EDR_*w*_) can be seen. This points to anisotropic turbulence as the cause of EDR scatter upstream of the mountain, where the atmosphere was stably stratified. For values of $\bar{\text{EDR}}\geq 0.05m^{2/3}s^{-1}$, the scatter is rather uniform, showing no general tendency of one EDR_*i*_ component prevailing over another. We conclude that the low EDR values should not be used, since the inertial dissipation technique cannot be applied in conditions of anisotropic turbulence. The scatter of EDR values exceeding 0.05 m^2/3^ s^−1^ is instead ascribed to statistical scatter and to possible shortcomings in the procedure of determining the inertial subrange. To obtain an estimate of the uncertainty in the measurement of EDR due to the scatter, we determine the percentage interval around $\bar{\text{EDR}}$ that includes 95% (‘±2*σ*’) of all ${\text{EDR}}_{i}\geq 0.05m^{2/3}s^{-1}$. The scatter for the flight legs from both days is almost identical and amounts to ±29% uncertainty of the individual EDR_*i*_ and ±16% uncertainty of the mean $\bar{\text{EDR}}$.

For the estimation of EDR_*w*_ from WCR data, two other possible sources of error have to be considered: the contamination of $\sigma_{v_{r}}^{2}$ by the horizontal wind and the deviation of the along‐track wind in a given radar range gate from the wind at flight level. The evaluation of these two terms is contained in Appendix 1.

For UWKA, the total uncertainty in the estimation of EDR is thus given by

(7) $$\text{EDR}=\bar{\text{EDR}}\pm 16\%$$

and for WCR, according to Appendix 1, by

(8) $$\begin{array}{lll} \text{EDR}= & ({{\text{EDR}}_{w}}_{-11\%}^{+9\%})\pm 29\%\; & \;\\ = & \left\{\begin{array}{cc} {{\text{EDR}}_{w}}_{-23\%}^{+41\%} & \text{for a tailwind},\\ {{\text{EDR}}_{w}}_{-37\%}^{+15\%} & \text{for a headwind}. \end{array}\right.\; & \end{array}$$

Note that the UWKA EDR estimate is based on three wind components, while that from WCR is based only on *w*. This is reflected in the uncertainty bounds.

## 4 Case studies

In this section, we analyse the spatial distribution of turbulence and its intensity across the MBM on three days of the NASA06 campaign. The impact of wave forcing on low‐level turbulence on 26 January and 5 February is contrasted with in‐cloud turbulence on 2 February. Table 1 lists the flight legs from these days.

**Table 1:** List of NASA06 research flight legs discussed in this article.

Date	Leg	Time	Altitude	Heading
(2006)		(UTC)	(m)	(°)
26 January	3	2121–2131	5200	101
	5	2154–2208	5180	100
5 February	1	1354–1404	5150	128
	2	1407–1423	4270	319
	3	1442–1451	4220	129
	4	1454–1508	4220	318
2 February	2	1915–1924	4250	101

### 4.1 *26 January 2006 –a hydraulic‐jump‐type rotor*

26 January 2006 represents the day of NASA06 with the strongest mesoscale dynamic forcing. Detailed observational analysis by FHO15 and numerical modelling by GSS15 have shown that this day can be characterized as a complex mountain flow event involving a large breaking wave in the mid‐troposphere, strong downslope winds over the lee slope of the mountain and a *hydraulic‐jump‐type* rotor (Lester and Fingerhut, 1974) which rapidly moved upstream.

**Figure 6:**
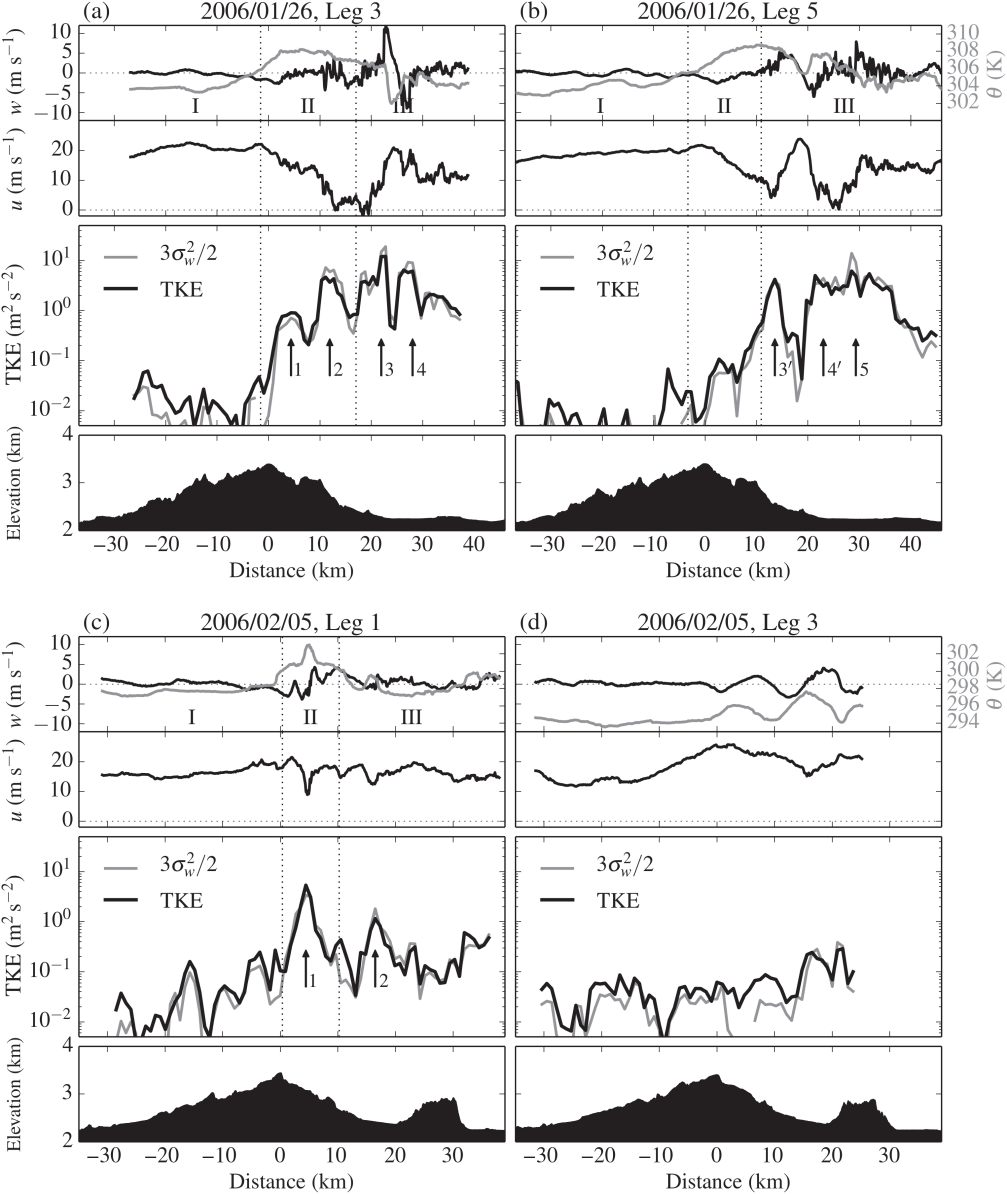
Aircraft *in situ* measurements of vertical velocity *w* (black solid), potential temperature *θ* (grey solid), along‐track horizontal velocity *u*, TKE and $3\sigma_{w}^{2}/2$, and the underlying terrain along flight legs crossing the MBM on (a,b) 26 January and (c,d) 5 February 2006. Vertical dotted lines plus letters I–III delimit distinct regions of the flow at flight level, discussed in section 4. Vertical arrows mark the location of peaks in turbulence intensity (also see Figure 4).

Figures 4(a,b) and 6(a,b) show UWKA measurements of *w*, *θ*, *u*, and TKE and EDR from two flight passes on 26 January, revealing the rapid evolution of the event. In Figure 6(a), corresponding to Leg 3, three distinct regions of the flow can be identified from flight‐level measurements. Region I upstream of the mountain peak is characterized by approximately constant along‐track horizontal wind *u*, zero vertical motion *w* and constant potential temperature *θ*. At the downstream end of Region I, a transition to downward motion and increasing *θ* is evident, which is associated with the descending branch of the wave. Region II exhibits increased but approximately constant *θ*, rapidly varying *w* and a sudden decrease of *u* from approx. 20 to 0 m s^−1^ and even negative values. Region III is characterized by a sudden, strong updraught and a slightly weaker downdraught on the order of +12 and −9 m s^−1^, respectively. The updraught coincides with a strong negative *θ* anomaly and re‐strengthened *u*. The changes in *u*, *w*, and *θ* along the leg are also reflected in TKE and EDR. Turbulence is ‘light’ (TKE and $\bar{\text{EDR}}$ below 0.06 m^2^ s^−2^ and 0.05 m^2/3^ s^−1^, respectively) in Region I and sharply increases as the aircraft penetrates into Region II (cf. Peaks 1 and 2). The strongest turbulence (TKE≃12.1 m^2^ s^−2^ and EDR≃0.38 m^2/3^ s^−1^) is found in Region III, collocated with the main up‐ and downdraught (Peaks 3 and 4). The two peaks are separated by a region of ‘light’ turbulence (TKE < 0.6 m^2^ s^−2^ and EDR < 0.05 m^2/3^ s^−1^).

Figure 6(b), corresponding to Leg 5 passing over the MBM around 25min later, shows a similar pattern in *w*, *θ* and *u*, but the main features in *w* and TKE have weakened considerably. Also notable is the upstream shift of the main updraught–downdraught couplet by approx. 7 km. Another remarkable change from Leg 3 to Leg 5 is the clear absence of turbulence in Region II preceding the updraught.

**Figure 7:**
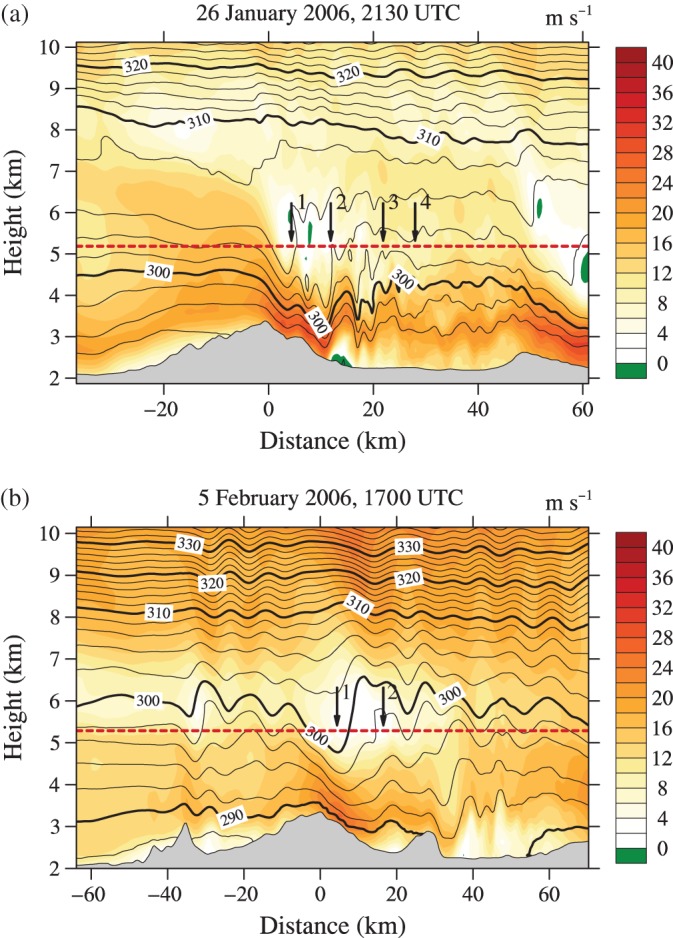
Numerical model cross‐sections showing along‐track horizontal wind component and isentropes. (a) Cross‐section along Leg 3 on 26 January for model output time 2130 UTC, indicating reversed flow along the lee slope close to the ground and steepened isentropes and stagnant flow at flight level. (b) Cross‐section along Leg 1 on 5 February for model output time 1700 UTC, providing a similar picture, but without indication of a near‐surface recirculation region. Note that the model output time of 1700 UTC has been chosen to account for the observed delayed timing of the model for the 5 February case (GSS15). Dashed horizontal lines indicate the altitude of the straight‐and‐level flight legs. Vertical arrows in both panels mark the location of peaks in turbulence intensity at flight level (Figure 6).

More insight into the dynamics behind the observed evolution of the event has been gained by GSS15 through high‐resolution numerical modelling with the Weather Research and Forecasting (WRF) model, using a horizontal grid spacing of 400 m. Model cross‐sections at the time of the observations show that the measurements along Legs 3 and 5 were taken during a period of ceasing wave breaking at mid‐tropospheric levels and consequent upstream shift of the rotor (GSS15, their Figures 8 and 11). One of the model cross‐sections is shown in Figure 7(a). Underneath the wave‐breaking region, a shooting flow over the lee of the MBM formed and was terminated by an internal hydraulic jump approx. 13 km downstream of the MBM top. However, the model hydraulic jump is positioned farther upstream than that encountered by the aircraft (approx. 22 km downstream of the MBM top). Regardless, the model helps link Region II in Figure 6(a) (and Peaks 1 and 2 in turbulence intensity) to mid‐level wave breaking. Despite the misplacement of the jump, the simulation also provides a hint at the origin of Peaks 3 and 4: at flight level, the aircraft encountered the upper edge of the hydraulic jump, giving rise to the strong up‐ and downdraught and a broad region of less coherent, but very turbulent, air motion downstream of it.

**Figure 8:**
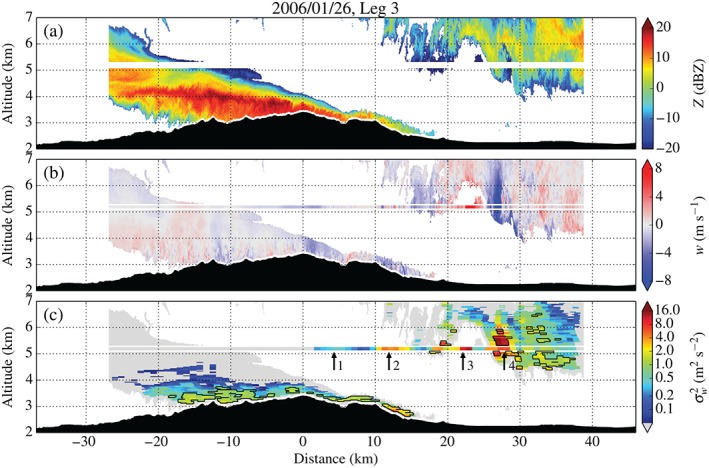
Radar data and derived turbulence parameters collected along Leg 3 on 26 January, crossing the MBM at approx. 5200 m amsl between 2121 and 2131 UTC. (a) Radar reflectivity *Z*, (b) vertical Doppler velocity *w*, and (c) variance of vertical velocity $\sigma_{w}^{2}$. White areas denote missing data due to insufficient radar backscatter from clouds. The white stripe in (a) corresponds to the ‘radar blind zone’, extending across ±100 m around flight level, which is filled with aircraft *in situ* measurements in (b) and (c). Raw vertical Doppler velocity *w* has been corrected for a mean hydrometeor fall speed of 1 m s^−1^. A logarithmically scaled colour bar is used for $\sigma_{w}^{2}$. Grey areas in the display of $\sigma_{w}^{2}$ correspond to an undetectable level of turbulence (greater than 100% relative uncertainty). The black contour marks the region in which the relative uncertainty of the $\sigma_{w}^{2}$ estimate is lower than 25%. Arrows in (c) indicate local maxima in $\sigma_{w}^{2}$, corresponding to the peaks in TKE in Figure 6(a).

**Figure 9:**
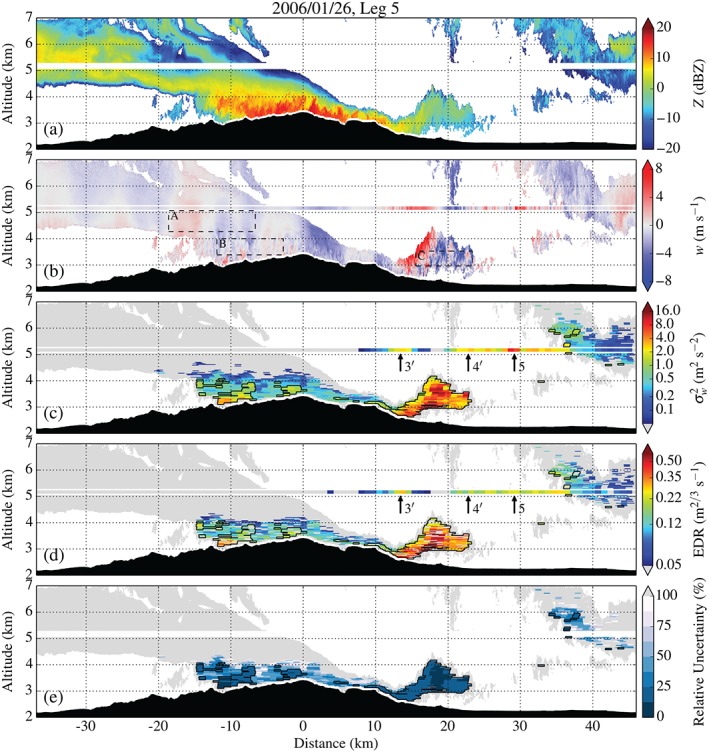
Radar data and derived turbulence parameters collected along Leg 5 on 26 January, crossing the MBM at approx. 5200 m amsl between 2154 and 2208 UTC. (a) Radar reflectivity *Z*, (b) vertical Doppler velocity *w*, (c) variance of vertical velocity $\sigma_{w}^{2}$, (d) dissipation rate EDR_*w*_, and (e) relative uncertainty of the $\sigma_{w}^{2}$ estimate. The black contour in (c)–(e) marks the region in which the relative uncertainty of the $\sigma_{w}^{2}$ estimate is lower than 25%. Arrows in (c) and (d) indicate local maxima in $\sigma_{w}^{2}$ and EDR, corresponding to the peaks in TKE in Figure 6(b). Boxes A–C in (b) delimit regions of the flow from which example radar spectra are shown in Figure 3(b).

Figures 8 and 9 show radar data and derived turbulence parameters along Legs 3 and 5. The first panel in both figures shows radar reflectivity *Z* which provides information on the distribution of cloud ice particles across the MBM. Due to poor radar coverage along Leg 3, only little additional insight can be gained from Figure 8, except for the dome‐like structure of the cloud distribution approx. 22 km downstream of the mountain top, marking the location of the hydraulic jump. Also note the good agreement between the variance estimates from the *in situ* and radar data right above and below the flight track between 26 and 38 km (Figure 8(c)), where radar backscatter was good. We proceed with the analysis of radar data from Leg 5, which provides better radar coverage.

Radar reflectivity in Figure 9(a) reveals a continuous layer of return signal of up to 3 km in depth upstream of the MBM and gradually decreasing depth in the downstream direction, reminiscent of compressed airflow in the accelerating downslope windstorm. At around 13 km downstream of the mountain top, the cloud layer is lifted from the surface and carried aloft to approx. 4.2 km amsl. The radar vertical Doppler velocity in Figure 9(b) reveals a strong updraught and a more diffuse downdraught (on the order of ±10 m s^−1^) within this region, associated with a large hydraulic‐jump‐type rotor extending over at least 1.5 km in the vertical (FHO15). Figure 9(c,d), showing the radar‐derived $\sigma_{w}^{2}$ and EDR_*w*_, provide insight into the near‐surface levels of turbulence. Note that the fields of EDR_*w*_ are generally patchier than those of $\sigma_{w}^{2}$, i.e. some radar data segments in the EDR plot remain grey while valid estimates were obtained for $\sigma_{w}^{2}$. This can be attributed to: (i) low turbulence, or (ii) the procedure of computing EDR (including the determination of the inertial subrange), which failed in some instances.

Upstream of and above the mountain top, turbulence decreases with distance from the ground, pointing to turbulence being shear‐generated. Turbulence is generally ‘light’ to ‘light‐moderate’ upstream of and above the MBM, but suddenly switches to ‘moderate‐severe’ and ‘severe’ at around 12 km down the lee slope. This sharp transition in turbulence intensity is collocated with the detachment of the boundary layer (FHO15) followed by a large rotor downstream of it. Maximum $\sigma_{w}^{2}$ and EDR_*w*_ in the separation region are 9.4 m^2^ s^−2^ and 0.51 m^2/3^ s^−1^, respectively. Highest turbulence intensities are detected inside the rotor. While the updraught itself appears ‘moderately’ turbulent, ‘severe’ turbulence is encountered in the downdraught region, with maximum $\sigma_{w}^{2}$ and EDR_*w*_ of 16.4 m^2^ s^−2^ and 0.77 m^2/3^ s^−1^, respectively. The relative uncertainty of $\sigma_{w}^{2}$, shown in Figure 9(e), remains within the 25% threshold in the separation region and inside the rotor. Unfortunately, the upstream side of the main updraught, which was detected to be most strongly turbulent in the *in situ* measurements (cf. Peak 3${}^{\prime }$), is missing in the radar data due to insufficient return signal.

### 4.2 *5 February 2006 –a lee‐wave rotor*

Observations and modelling of the 5 February 2006 case (FHO15 and GSS15) have shown that the flow on this day is characterized by large‐amplitude waves in the lee of the MBM and a *lee‐wave* rotor (Lester and Fingerhut, 1974) with some degree of transitional behaviour, displaying a similar upstream movement as that of the 26 January case.

Our focus is again on the spatial distribution and magnitude of turbulence. Figures 4(c,d) and 6(c,d) show UWKA measurements of *w*, *θ*, *u*, and TKE and EDR from Legs 1 and 3 on 5 February. Unlike the passes flown on 26 January, legs on this day were not flown at the same altitude (cf. Table 1). We again subdivide measurements along Leg 1 into Regions I–III. Region I bears some resemblance with that on 26 January. Along‐track horizontal wind *u* is approximately constant, while *w* transitions from near zero to negative (−3 m s^−1^) at the downstream end of Region I, where *θ* increases rapidly by approx. 3 K. This again points to the fact that UWKA was first sampling in the relatively undisturbed upstream environment and then penetrated into a wave trough downstream of Region I. Region II is characterized by stronger variations in *w*, a drop in *u* by around 10 m s^−1^, and a remarkably sharp, localized increase in *θ* of another 2 K. This feature coincides with the strongest peak (Peak 1 in Figures 4(c) and 6(c)) in TKE and $\bar{\text{EDR}}$ of 5.4 m^2^ s^−2^ and 0.25 m^2/3^ s^−1^, respectively. Region III lacks the strong updraught and downdraught couplet present along Leg 3 of 26 January but has a secondary positive *θ* anomaly of approx. 2 K and local minimum in *u* collocated with a local turbulence maximum (Peak 2), whose origin, however, is not entirely understood.

The presence and origin of Peak 1 in turbulence intensity has remained unaddressed in previous studies. Closer inspection of a model cross‐section from 5 February (Figure 7(b)) indicates a relatively warm and stagnant region of air at and above flight level slightly downstream of the mountain top, whose location exactly matches the observed *θ* and turbulence maximum. Thus, it is plausible that the observations made along Leg 1 of 5 February document another aircraft encounter with a wave‐breaking region. UWKA measurements along Leg 3 of that day largely differ from those along Leg 1, owing to the fact that the aircraft crossed the MBM at approx. 1 km lower altitude. The variation of *w* and *θ* along Leg 3 evidences a smooth lee wave. Turbulence intensities remain below 0.3 m^2^ s^−2^ and 0.07 m^2/3^ s^−1^.

**Figure 10:**
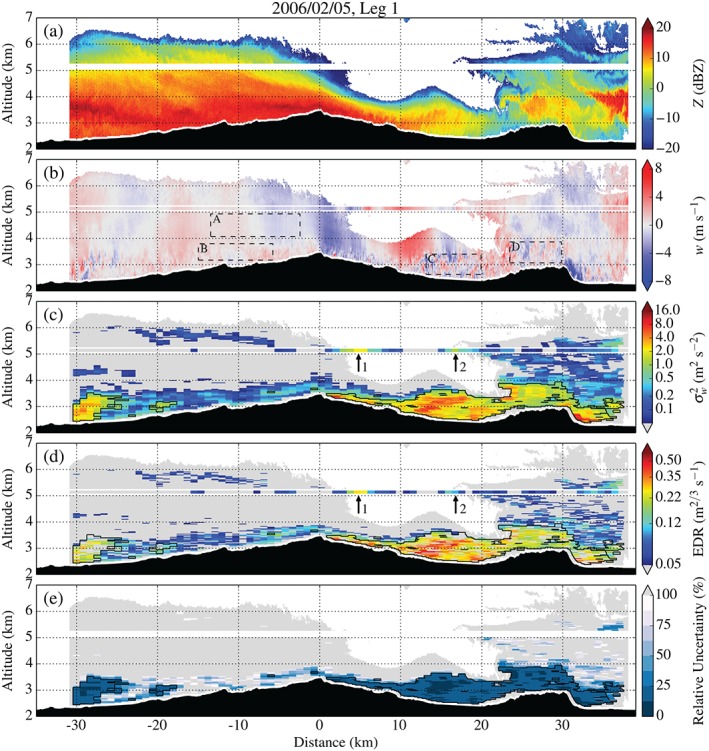
As Figure 9, but for Leg 1 on 5 February, crossing the MBM and Sheep Mountain at approx. 5150 m amsl between 1354 and 1404 UTC. Boxes A–D in (b) delimit the regions of the flow of which example radar spectra are shown in Figure 3(d). Arrows in (c) and (d) indicate local maxima in $\sigma_{w}^{2}$ and EDR, corresponding to the peaks in TKE in Figure 6(c).

**Figure 11:**
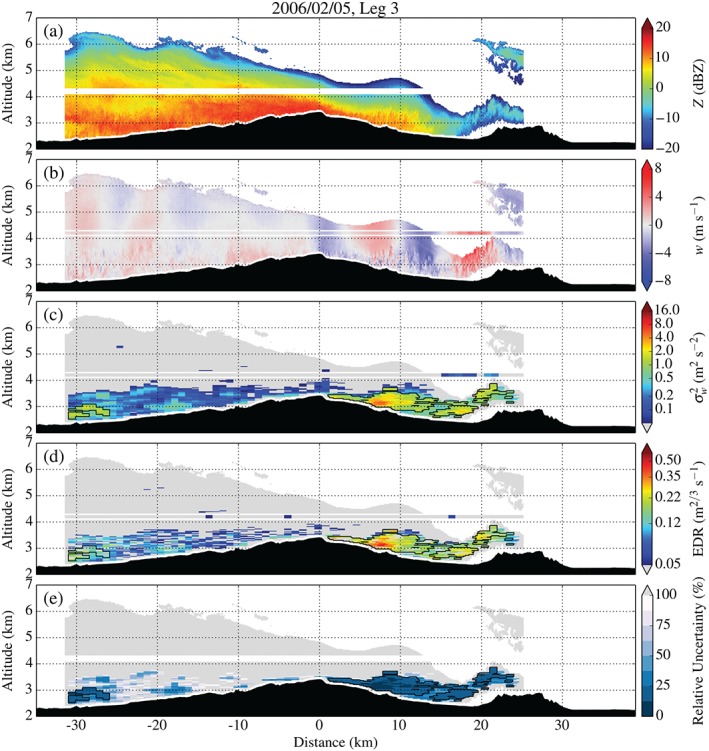
As Figure 9, but for Leg 3 on 5 February, crossing the MBM and Sheep Mountain at approx. 4200 m amsl between 1442 and 1451 UTC.

Figures 10 and 11 show radar data and derived fields along Legs 1 and 3. Leg 1 of 5 February is the leg with the best radar coverage of all legs on 26 January and 5 February. Above the lee slope of the MBM, radar vertical Doppler velocities reveal strong descending motion (−4 m s^−1^), followed by upward (+4.5 m s^−1^) and weaker downward (−2 m s^−1^) motion, associated with a lee wave. Right below the lee‐wave crest, speckled positive and negative vertical velocities indicate enhanced levels of turbulence. Using dual‐Doppler analysis, FHO15 detected near‐surface reversed flow in this region, revealing the presence of a lee‐wave rotor. Small‐scale variations in *w*, indicative of turbulence, extend farther downstream towards the upwind slope of Sheep Mountain. A strong downdraught (up to −8 m s^−1^) is detected on the lee side of Sheep Mountain. Radar‐derived turbulence intensities reveal several regions of ‘moderate’ to ‘severe’ turbulence upstream and downstream of the MBM. The rather large patch of turbulence on the western edge of the stretch can be attributed to turbulence generated at the flanks of Elk Mountain (Karacostas and Marwitz, 1980), which is located approx. 35 km upstream of the MBM (cf. Figure 1).

Turbulence on the lee side of the MBM exhibits a complex spatial structure. A small patch of ‘moderate‐severe’ turbulence can be found in the immediate lee of Medicine Bow Peak (1–3 km downstream of it), with maximum turbulence intensities $\sigma_{w}^{2}$ and EDR_*w*_ of 10.4 m^2^ s^−2^ and 0.41 m^2/3^ s^−1^, respectively. Such strong turbulence right behind a steep peak is often associated with *bluff‐body separation* of the boundary layer, occurring even without wave forcing from aloft (Baines, 1997). The largest region of strong turbulence is found in the rotor, beneath the lee‐wave crest. Turbulence is strongest in the westernmost part of the rotor, with maximum turbulence intensities reaching 7.8 m^2^ s^−2^ and 0.50 m^2/3^ s^−1^. In the region of strong downslope winds in the lee of Sheep Mountain a maximum in $\sigma_{w}^{2}$ of 9.5 m^2^ s^−2^ is detected, but no reliable estimate of EDR is available.

In the analysis of UWKA measurements, we pointed to the two peaks (Peak 1 and 2) in TKE at flight level and briefly discussed the possible origin of Peak 1. The variance $\sigma_{w}^{2}$ from *in situ* and radar measurements reveals that turbulence at flight‐level is clearly separated from strong turbulence closer to the ground by a region of low (i.e. undetectable) turbulence (grey colour in Figure 10(c,d)). This underpins the distinct origins of turbulence in these regions and, indirectly, supports the conjecture of mid‐tropospheric wave breaking on 5 February.

Figure 11 contains the radar turbulence analysis for Leg 3, crossing the MBM around 40min later. The first lee‐wave crest has moved upstream and a train of waves (apparent from the combined UWKA and WCR measurements of *w* in Figure 11(b)) has formed. In response to the relocation of the first lee‐wave crest, the region of strong turbulence has retreated upstream, decreased in size and weakened somewhat (maximum turbulence intensities around 5.7 m^2^ s^−2^ and 0.45 m^2/3^ s^−1^).

**Figure 12:**
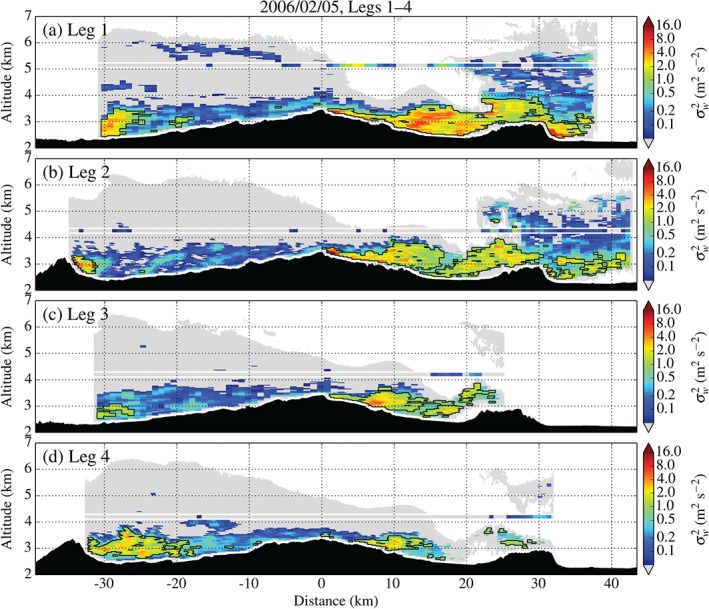
Variance of vertical Doppler velocity $\sigma_{w}^{2}$ for (a–d) Legs 1–4 on 5 February, crossing Elk Mountain, the MBM, and Sheep Mountain between 1354 and 1508 UTC. Note that all flight legs crossed the peak region of the MBM but Legs 2 and 4 were flown at a somewhat larger heading (approx. +10°), thereby passing over the top of Elk Mountain at the western end of the transect.

The evolution of the 5 February event is further evidenced in Figure 12 by the composite analysis of Legs 1–4. The gradual retreat of the location of turbulence on 5 February bears some resemblance with the upstream shift of the rotor and its turbulence on 26 January. Unfortunately, along Legs 2–4, no data at the flight level of Leg 1 (5150 m amsl) is available. Thus, observational evidence of the evolution of gravity‐wave breaking is lacking and, judging from the aircraft measurements alone, we can only speculate about the reason for the ceasing and retreating rotor turbulence. However, model cross‐sections on 5 February after 1630 UTC (GSS15, their Figure 12) do support the idea that the evolution of the flow at mid‐levels, i.e. the onset and cessation of wave breaking, is the likely cause of the upstream shift of the rotor, as with the 26 January event. Note that the model runs for this case did reproduce the upstream shift too, although with a time delay of 3–4 h.

### 4.3 *2 February 2006—a reference case with no indication of gravity waves*

For comparison, we also include the analysis of radar data collected along one cross‐mountain pass on 2 February 2006. Upstream conditions on this day were characterized by moderate mean wind speed and considerably lower static stability relative to the two wave cases (Geerts *et al.* 2011). No gravity waves were excited by the MBM on this day.

**Figure 13:**
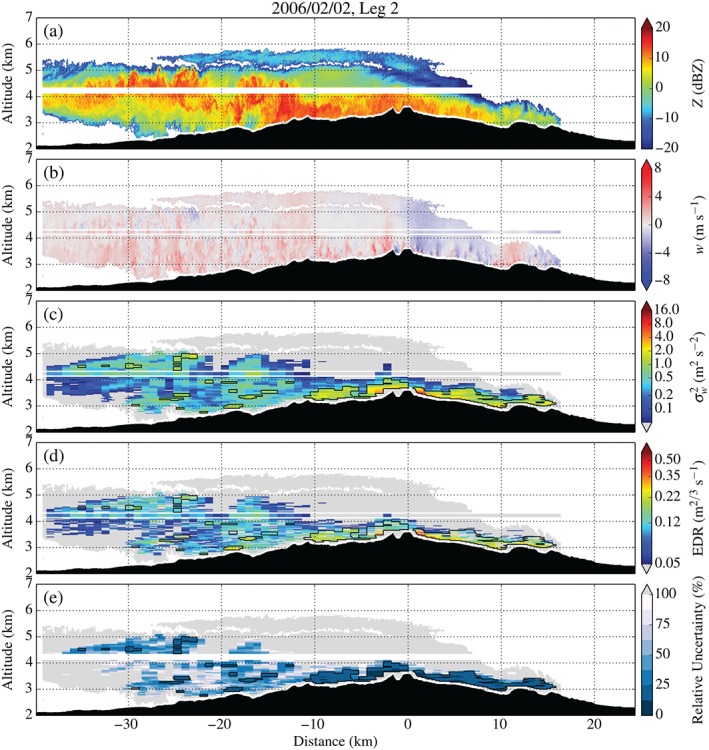
As Figure 9, but for Leg 2 on 2 February crossing the MBM at approx. 4250 m amsl between 1915 and 1923 UTC.

Figure 13 shows radar data collected along Leg 2 of 2 February. The upstream part of the flow exhibits ‘light‐moderate’ turbulence ($\sigma_{w}^{2}$ and EDR_*w*_ up to 1.8 m^2^ s^−2^ and 0.18 m^2/3^ s^−1^, respectively). Right upstream of the mountain top, ‘moderate’ turbulence (up to 3.9 m^2^ s^−2^ or 0.32 m^2/3^ s^−1^) is detected in a region of enhanced vertical air motion. ‘Moderate‐severe’ turbulence (up to 6.7 m^2^ s^−2^ or 0.43 m^2/3^ s^−1^) is detected in the immediate lee of a steep peak at mountain top, as with Leg 1 on 5 February.

## 5 Turbulence intensities in mountain‐induced turbulent processes

In the previous section, detailed analysis of three distinct mountain flow cases has revealed a variety of mountain‐induced turbulent processes, ranging from in‐cloud turbulence to wave‐induced boundary‐layer separation and rotor formation. Table 2 summarizes the turbulence intensities for each of the phenomena studied in this work, including the measurement uncertainties. Table 3 lists observations of the same phenomena documented in the literature. The comparison of our results to turbulence intensities reported in the literature reveals the sensitivity of turbulence measures to the chosen filter scale. It is worth stressing that, for the cases considered in this work, the choice of a filter scale of 1.5 km was made on the basis of high‐pass filtering tests which remove apparent features of mesoscale motion from the spatial series, leaving us with the turbulent component of the signal.

**Table 2:**
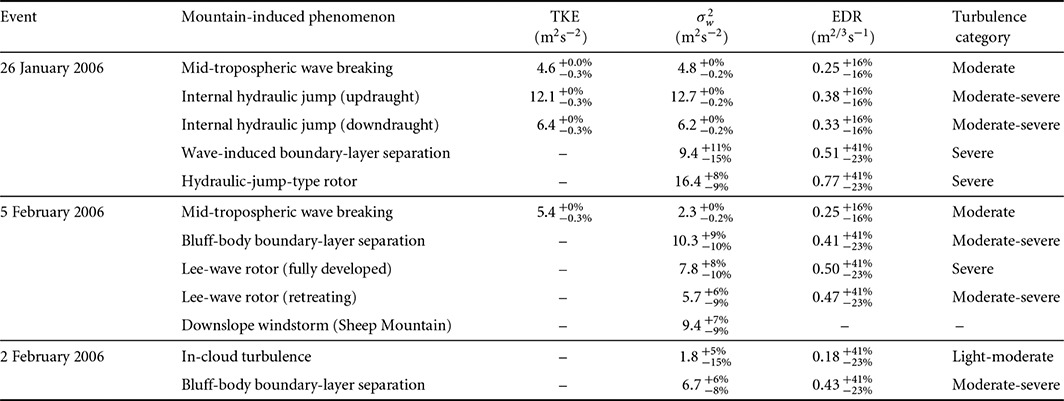
List of turbulent phenomena encountered on three days of NASA06 and associated maximum turbulence intensities, including their upper and lower uncertainty bounds (referred to as *relative uncertainty R* in the text, cf. Eq. (3)). The last column provides the associated *turbulence category* used in pilot reports (PIREPs) in civil aviation. Turbulence categories ‘smooth‐light’, ‘light’, ‘light‐moderate’, ‘moderate’, ‘moderate‐severe’, and ‘severe’ correspond, respectively, to EDR thresholds of 0.014, 0.050, 0.125, 0.220, 0.350, 0.500 m^2/3^ s^−1^ (Sharman *et al.* 2014).

**Table 3:** Summary of past aircraft observations of mountain‐induced turbulent phenomena, documented by (a) Lilly (1978), (b) Jiang and Doyle (2004), (c) Smith (1987), (d) Elvidge *et al.* (2014), (e) Armi and Mayr (2011), (f) Lester and Fingerhut (1974), (g) Darby and Poulos (2006) and (h) Cohn *et al.* (2011).

Mountain‐induced	Case	Mountain range	Reported turbulence	Filter
phenomenon			intensity	scale (km)
Mid‐tropospheric	11 January 1972 (a)	Front Range, CO	TKE≃ 150m^2^ s^−2^	18
gravity‐wave	21 October 1999 (MAP) (b)	Central Alps, Austria	TKE≃ 10.5m^2^ s^−2^	5
breaking	7 March 1982 (ALPEX) (c)	Dinaric Alps, Croatia	$\sigma_{w}^{2}≃$ 5–10m^2^ s^−2^	1.26
	5 February 2011 (d)	Antarctic Peninsula	TKE≃ 5–9m^2^ s^−2^	3
Internal	7 March 1982 (ALPEX) (c)	Dinaric Alps, Croatia	$\sigma_{w}^{2}≃$ 10–15m^2^ s^−2^	1.26
hydraulic jump	9 April 2006 (T‐REX) (e)	Sierra Nevada, CA	EDR≃ 0.35 m^2/3^ s^−1^	—
Atmospheric	8 October 1970 (f)	Front Range, CO	$\sigma_{u}^{2}≃$ 2.6m^2^ s^−2^	0.85
rotors			$\bar{\text{EDR}}≃$ 0.19–0.34 m^2/3^ s^−1^	—
	1 April 1997 (g)	Front Range, CO	EDR≃ 0.33m^2/3^ s^−1^	—
	9 March 2006 (T‐REX) (h)	Sierra Nevada, CA	EDR≃ 0.21m^2/3^ s^−1^	—

### 5.1 *Mid‐tropospheric gravity‐wave breaking*

Direct observations of mid‐tropospheric gravity‐wave breaking through aircraft encounters are quite rare. We are aware of only four studies examining direct aircraft measurements of turbulent wave breakdown at mid‐levels. Lilly (1978) investigated the ‘mid‐tropospheric turbulence zone’ during the Boulder windstorm of 11 January 1972. A case of gravity‐wave breaking over the Central Alps during MAP on 21 October 1999 was documented by Jiang and Doyle (2004). Elvidge *et al.* (2014) described low‐level wave breaking at a wave‐induced critical level during a föhn event over the Antarctic Peninsula. A case of severe Bora flow on the Adriatic coast of Croatia was observed on 7 March 1982 during ALPEX (Smith, 1987). Figure 6b of Smith (1987) shows the breakdown of a large‐amplitude gravity wave at mid‐levels (approx. 3000 m amsl), roughly 2000 m above the top of the Dinaric Alps. Underneath the turbulent breakdown region of the ALPEX case, steepened isentropes evidence a hydraulic‐jump‐like feature. Turbulence estimates from the latter two studies compare best with our estimates of TKE in regions of wave breaking and hydraulic jumps, in particular in the 26 January case.

### 5.2 *Internal hydraulic jumps*

Direct aircraft measurements in hydraulic jumps are also relatively rare. We are only aware of another study by Armi and Mayr (2011) documenting a jump‐like feature in the lee of the Sierra Nevada, California, on 9–10 April 2006, during Intensive Observing Period 11 of T‐REX. The maximum EDR value of 0.35 m^2/3^ s^−1^ reported for that event is in striking agreement with 0.38 m^2/3^ s^−1^ in the updraught part of the jump in the 26 January case, obtained in this study.

### 5.3 *Atmospheric rotors*

On 26 January and 5 February, rotor circulations were captured by WCR along several passes across the MBM, allowing the documentation of the quasi‐instantaneous spatial distribution and intensity of turbulence in the rotors' interior. Unfortunately, radar data from the rotor is incomplete for 26 January due to insufficient radar backscatter at lower levels. Nevertheless, turbulence intensities from Leg 5 (Figure 9) suggest that the strongest turbulence (of the ‘severe’ category) is located right downstream of the leading updraught of this hydraulic‐jump‐type rotor. On 5 February, a similar picture is obtained from Leg 1 passing over the fully‐developed lee‐wave rotor (Figure 10) and from Leg 3 (Figure 11) during its later stages. The detected spatial distribution of turbulence within the rotor is in agreement with previous observations by Lester and Fingerhut (1974) and Cohn *et al.* (2011).

Using numerical simulation and ground‐based remote sensing, Doyle and Durran (2002, 2007), and Doyle *et al.* (2009) studied the inner flow field of rotors and attributed patches of strong turbulence inside the rotor to intermittent smaller‐scale vortical structures with characteristic length‐scales of 500–1000 m. These ‘subrotors’ were found to originate from Kelvin–Helmholtz instability in the separated boundary layer. The presence of subrotors on 26 January and 5 February was confirmed by FHO15 considering the cross‐track vorticity along the rotor crest, derived from dual‐Doppler analysis. Owing to the chosen leg segment length, our turbulence analysis approach does not allow the subrotor structures to be resolved explicitly. However, separate patches of ‘moderate’ to ‘severe’ turbulence in the rotor interior are apparent from the fields of turbulence intensity and attributing these to subrotors seems a plausible explanation.

There are only a few observational studies providing quantitative estimates of turbulence intensity in a rotor. For instance, Lester and Fingerhut (1974) report approximate values for the variance of the longitudinal velocity $\sigma_{u}^{2}$ and EDR. Our results are also in good agreement with a more recent study by Darby and Poulos (2006), making use of UWKA measurements to study the evolution of lee‐wave rotor activity in the lee of Pikes Peak, Colorado.

### 5.4 *Bluff‐body boundary‐layer separation*

Another turbulent process covered by the radar observations is the bluff‐body separation of the boundary layer in the immediate lee of steep peaks. This phenomenon, occurring in both neutrally and stably stratified fluids, is well known in aeronautical engineering and has occasionally been observed in the atmosphere, e.g. in the Sierra Nevada during T‐REX (Grubišić *et al.* 2006; Haimov *et al.* 2008). Of the numerous turbulence‐related aviation incidents in the vicinity of mountains (Carney *et al.* 1995), some are likely related to the phenomenon.

Bluff‐body boundary‐layer separation was observed on 2 February and 5 February downstream of Medicine Bow Peak, with ‘moderate‐severe’ maximum $\sigma_{w}^{2}$ and EDR_*w*_ in the range 6.7–10.3 m^2^ s^−2^ and 0.41–0.43 m^2/3^ s^−1^, respectively.

## 6 Summary and conclusions

Airborne *in situ* and single‐Doppler radar measurements over the Medicine Bow Mountains in southeast Wyoming have been used to study atmospheric turbulence generated in flow over mountainous terrain. Measurements during several complex mountain flow cases were conducted by UWKA and WCR during the NASA06 campaign. The collected data offers the opportunity to study a variety of turbulent mountain flow phenomena ranging from gravity‐wave breaking to atmospheric rotors.

Our analysis focuses on describing the spatial distribution of turbulence and on providing quantitative estimates of turbulence intensity, in terms of turbulent kinetic energy (TKE), variance of vertical velocity ($\sigma_{w}^{2}$) and cube root of the energy dissipation rate (EDR). The main findings of this study are twofold, pertaining to the turbulence measurement technique and to the quantification of atmospheric turbulence over complex terrain.

Prior to this study, it was not clear whether the inherent inaccuracies in measured single‐Doppler velocities from airborne fixed‐antenna radar related to the motion of the measurement platform would allow quantitative turbulence estimates in spatially inhomogeneous airflow. By carrying out a thorough analysis of potential sources of error in the Doppler wind retrieval and their effect on the uncertainty of the turbulence estimates, we have shown here that this question can be answered in the affirmative.

In the estimation of the variance of vertical air motion from the radar, the following sources of uncertainty needed to be taken into account: (i) Uncertainty in the determination of aircraft motion and attitude; (ii) limited accuracy of the beam pointing‐angle calibration; (iii) variance of hydrometeor fall speed; (iv) loss of variance due to the radar pulse‐volume‐averaging effect; and (v) contamination of radar Doppler velocity by the horizontal wind.

For the analysed cases, the sum of these terms remains sufficiently small to allow a quantitative measurement of turbulence with the airborne Doppler radar. While only qualitative estimates of turbulence intensity can be obtained outside the most turbulent regions, 25% accuracy and better is achieved in regions of ‘moderate’ to ‘severe’ turbulence in the lee of the mountains. The minimum detectable turbulence in the cases under consideration is limited to approximately 0.1 m^2^ s^−2^ and 0.05 m^2/3^ s^−1^ for $\sigma_{w}^{2}$ and EDR, respectively. However, turbulence estimates are reliable, i.e. affected by reasonably small uncertainty levels, only for values greater than 0.5 m^2^ s^−2^ and 0.15 m^2/3^ s^−1^ for $\sigma_{w}^{2}$ and EDR, respectively. It is also worth pointing to the good agreement between *in situ* and radar‐derived turbulence estimates, which is apparent, for example, in Figures 8, 9, and 13. While detailed comparisons between *in situ* and remotely sensed turbulence estimates have been made in other studies (e.g. Istok and Doviak, 1986; Meischner *et al.*, 2001; Lothon *et al.*, 2005), a systematic analysis using the NASA06 data set is not possible due to generally poor radar backscatter right above and below flight level.

The thresholds of minimum detectable turbulence and uncertainty provided above strictly apply to the data under consideration here. Future application of the analysis technique to a different data set will necessitate careful re‐evaluation of each of the terms contributing to the uncertainty. Some of these terms are tightly linked to properties of the instruments used (for instance, the aircraft motion correction or the pulse‐volume‐averaging effect) and their evaluation can largely follow what has been proposed in this work. However, special attention has to be dedicated to the uncertainties associated with environmental parameters, which are expected to display a large variability from case to case. This applies in particular to the contamination of vertical Doppler velocity by the horizontal wind and the variance of hydrometeor fall speed, the latter of which was found to considerably limit the quality of results in other cases (Lothon *et al.* 2005).

Two days of the NASA06 campaign with strong gravity‐wave forcings have been analysed. Results are summarized in Table 2, providing maximum turbulence intensities and relative uncertainties for each observed mountain‐induced phenomenon. On both days, a region of turbulent breakdown of a large gravity wave is observed approx. 1600 m above mountain top; ‘moderate’ turbulence is detected in the breaking regions (maximum TKE and EDR of 5.4 m^2^ s^−2^ and 0.25 m^2/3^ s^−1^, respectively). These observations add to barely a handful of observations of mid‐tropospheric gravity‐wave breaking documented in the literature.

A unique result of this study is the quantitative estimation of the intensity of turbulence and its spatial distribution in the interior of atmospheric rotors, provided by the radar‐derived turbulence fields. Maximum turbulence intensities in both the hydraulic‐jump‐type rotor and the lee‐wave‐type rotor are detected below the updraught of the main wave aloft, in agreement with past observations and numerical studies of rotor turbulence. In both cases, turbulence in the rotor is ‘severe’, with $\sigma_{w}^{2}$ and EDR in the ranges 7.8–16.4 m^2^ s^−2^ and 0.50–0.77 m^2/3^ s^−1^, respectively. The spatial distribution of turbulence maxima inside the rotor seems patchy, reminiscent of individual smaller‐scale vortices (subrotors; Doyle *et al.*, 2009). Apart from wave‐induced processes, ‘moderate‐severe’ turbulence (6.7–10.3 m^2^ s^−2^ and 0.41–0.43 m^2/3^ s^−1^) is also detected leeward of steep peaks, underlining the threat for aircraft approaching mountain slopes too closely.

In conclusion, it has been demonstrated that combined aircraft *in situ* and Doppler radar measurements allow the documentation of mountain‐induced turbulence at unprecedented spatial resolution and reasonable accuracy. The compiled list of mountain‐induced phenomena and associated turbulence intensities (Table 2) including civil aviation turbulence categories, can provide updated turbulence reference values for these phenomena to the community of aviation weather forecasters. In this context, it is hoped that this study can further the knowledge of the hazards involved in flying in the vicinity of mountains, thereby contributing to improved turbulence avoidance strategies.

## Figures and Tables

**Figure A1:**
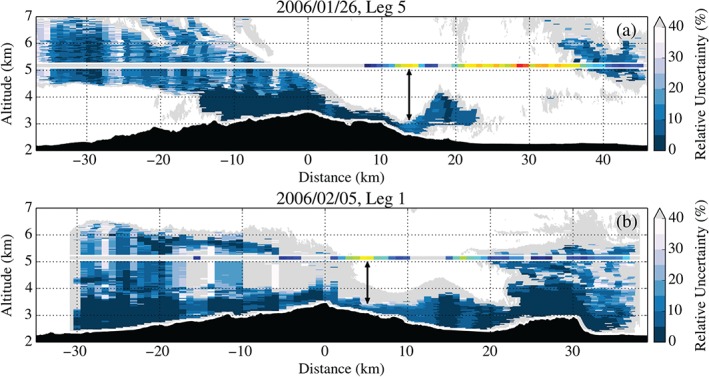
Relative uncertainty of the variance of vertical velocity $\sigma_{w}^{2}$ due to the contamination by the horizontal wind for (a) Leg 5 on 26 January, and (b) Leg 1 on 5 February. Coloured stripes, inserted at flight level, correspond to *in situ* measured $\sigma_{w}^{2}$ (cf. Figures 9(c) and 10(c)). Vertical arrows indicate the collocation of enhanced flight‐level turbulence and increased relative uncertainty in the estimation of $\sigma_{w}^{2}$ in the lower‐level flow.

## Footnotes

* Hereafter FHO15 and GSS15, respectively.

## Appendix A 1

### Uncertainty in the variance of vertical velocity $\sigma_{w}^{2}$ due to contamination by the horizontal wind

Along the NASA06 flight legs under consideration, UWKA encountered moderate turbulence at flight level, leading to considerable deviations from mean pitch and zero roll and drift angles. As a result, the fixed radar beams were not pointing perfectly vertically, causing a possible contamination of the radar‐measured vertical air velocity by the horizontal wind. It is not possible to accurately correct for this contamination since the along‐track wind in a given radar range deviates up to 30 m s^−1^ from the one at flight altitude but is not known exactly. In this section, we evaluate the effect of the contamination on the uncertainty of the variance of vertical velocity $\sigma_{w}^{2}$.

To estimate the contamination, we relate the components of the wind $\tilde{u},\tilde{v}$, and $\tilde{w}$ in the aircraft‐fixed reference system (ACRS) to the components *u*,*v*, and *w* in the along‐track reference system (ATRS). In the ACRS, the unit vector **e**_*x*_ is parallel to the aircraft longitudinal axis, ${\tilde{e}}_{y}$ is 90° to the right of the longitudinal axis, and ${\tilde{e}}_{z}$ is pointing down. In the ATRS, **e**_*x*_ points in the along‐track direction, **e**_*y*_ points 90° horizontally right from it in the cross‐track direction, and **e**_*z*_ points nadir.

The velocity vector of an air parcel **v** can be expressed as

(A1) $$v=\tilde{u}{\tilde{e}}_{x}+\tilde{v}{\tilde{e}}_{y}+\tilde{w}{\tilde{e}}_{z}=ue_{x}+ve_{y}+we_{z}.$$

The components of **v** in the ATRS and the ACRS are related by a transformation, given by a series of rotations. Following author=Wendisch, M (2013), we use the Tait–Bryan sequence of rotations: (i) rotation **R** about ${\tilde{e}}_{x}$ by roll angle (*φ*, right wing down positive); (ii) rotation **P** about ${\tilde{e}}_{y}$ by pitch angle (*θ*, nose up positive); and (iii) rotation **D** about ${\tilde{e}}_{z}$ by drift angle (*γ*, positive in clockwise direction), with the rotation matrices being defined as

(A2) $$\begin{array}{lll} R\; & =\;\;\left(\begin{array}{lll} 1 & 0 & 0\\ 0 & \cos\varphi & -\sin\varphi \\ 0 & \sin\varphi & \cos\varphi \end{array}\right)\;\;,P\;=\;\;\left(\begin{array}{lll} \cos\theta & 0 & \sin\theta \\ 0 & 1 & 0\\ -\sin\theta & 0 & \cos\theta \end{array}\right)\;\;,\; & \;\\ D\; & =\;\;\left(\begin{array}{lll} \cos\gamma & -\sin\gamma & 0\\ \sin\gamma & \cos\gamma & 0\\ 0 & 0 & 1 \end{array}\right).\; & \end{array}$$

The transformation from the ACRS to the ATRS is then given by

(A3) $${\left(\begin{array}{lll} u & v & w \end{array}\right)}^{t}=DPR{\left(\begin{array}{lll} \tilde{u} & \tilde{v} & \tilde{w} \end{array}\right)}^{t}.$$

Inverting this equation for the vertical component in the ACRS $\tilde{w}$ and identifying $\tilde{w}$ with the radial Doppler velocity *v*_r_, we get the expression for the contamination by the horizontal wind as

(A4) $$\begin{array}{lll} v_{r}=\tilde{w}= & u(\sin\gamma \sin\varphi +\sin\theta \cos\gamma \cos\varphi )\; & \;\\ & +v(\sin\gamma \sin\theta \cos\varphi -\sin\varphi \cos\gamma )\; & \;\\ & +w\cos\varphi \cos\theta .\; & \end{array}$$

Approximating sines and cosines for small aircraft attitude angles (maximum *φ*,*θ*,*γ*≤ 6°≃0.11 rad), and neglecting products of small angles, this can be simplified to a good approximation to

(A5) $$v_{r}=\tilde{w}=\mathrm{u\theta}-\mathrm{v\varphi}+w.$$

To relate the variance of radial Doppler velocity $\sigma_{v_{r}}^{2}$ and the variance of vertical velocity $\sigma_{w}^{2}$, all relevant quantities are split into their mean and fluctuation parts ($x=\bar{x}+x^{\prime }$; *x*={*u*,*v*,*w*,*v*_r_,*φ*,*θ*}), to be evaluated along individual leg segments.

Inserting these into Eq. (A5) and averaging leads to

(A6) $$\begin{array}{lll} & \bar{{\left(\bar{v_{r}}+v_{r}^{{}^{\prime }}\right)}^{2}}\; & \;\\ & =\bar{{\left[\left(\bar{\theta }\;+\;\theta^{{}^{\prime }})(\bar{u}\;+\;u^{{}^{\prime }})\;-\;(\bar{\varphi }\;+\;\varphi^{{}^{\prime }})(\bar{v}\;+\;v^{{}^{\prime }})\;+\;(\bar{w}\;+\;w^{{}^{\prime }}\right)\right]}^{2}}.\; & \end{array}$$

Expanding Eq. (A6) results in a total of 55 terms involving first‐ to fourth‐order moments of the perturbation variables. The number of terms can be reduced substantially by making the following simplifications and assumptions:

- $\bar{v_{r}}=\bar{u}\bar{\theta }-\bar{v}\bar{\varphi }+\bar{w}$.
- $\bar{v_{r^{{}^{\prime }}}}=\bar{u^{{}^{\prime }}}=\bar{v^{{}^{\prime }}}=\bar{w^{{}^{\prime }}}=\bar{\theta^{{}^{\prime }}}=\bar{\varphi^{{}^{\prime }}}=0$.
- Roll and pitch angle perturbations {*θ*′,*φ*′} are uncorrelated with wind fluctuations {*u*′,*v*′,*w*′}; e.g. $\bar{w^{{}^{\prime }}\theta^{{}^{\prime }}}$ = 0.
- Assuming that perturbations follow normal distributions, Isserlis’ theorem (Isserlis, 1918) applies to third‐ and fourth‐order moments; e.g.

$$\begin{array}{lll} \bar{\theta^{{}^{\prime }}u^{{}^{\prime }}v^{{}^{\prime }}} & =0\;\;\mathrm{and}\; & \\ \bar{\theta^{{}^{\prime }}\varphi^{{}^{\prime }}u^{{}^{\prime }}v^{{}^{\prime }}} & =\bar{\theta^{{}^{\prime }}\varphi^{{}^{\prime }}}\bar{u^{{}^{\prime }}v^{{}^{\prime }}}+\bar{\theta^{{}^{\prime }}u^{{}^{\prime }}}\bar{\varphi^{{}^{\prime }}v^{{}^{\prime }}}+\bar{\theta^{{}^{\prime }}v^{{}^{\prime }}}\bar{\varphi^{{}^{\prime }}u^{{}^{\prime }}}.\; & \; \end{array}$$

With $\sigma_{x}^{2}\equiv \bar{x^{{}^{\prime }2}}$ for the variance of a quantity *x*, we finally obtain an equation relating $\sigma_{v_{r}}^{2}$ to $\sigma_{w}^{2}$:

(A7) $$\begin{array}{lll} \sigma_{v_{r}}^{2}=\sigma_{w}^{2} & +\sigma_{\theta }^{2}{\bar{u}}^{2}+{\bar{\theta }}^{2}{\sigma_{u}}^{2}+\sigma_{\theta }^{2}{\sigma_{u}}^{2}\; & \;\\ & +\sigma_{\varphi }^{2}{\bar{v}}^{2}+{\bar{\varphi }}^{2}{\sigma_{v}}^{2}+\sigma_{\varphi }^{2}{\sigma_{v}}^{2}\; & \;\\ & +2\bar{\theta }\bar{u^{{}^{\prime }}w^{{}^{\prime }}}-2\bar{\varphi }\bar{v^{{}^{\prime }}w^{{}^{\prime }}}-2\bar{\theta^{{}^{\prime }}\varphi^{{}^{\prime }}}\bar{u^{{}^{\prime }}v^{{}^{\prime }}}\; & \;\\ & -2\bar{\theta^{{}^{\prime }}\varphi^{{}^{\prime }}}\bar{u}\bar{v}-2\bar{\theta }\bar{\varphi }\bar{u^{{}^{\prime }}v^{{}^{\prime }}}.\; & \end{array}$$

Terms 2–7 on the right‐hand side of Eq. (A7) consist of (positive semi‐definite) products of squares of the mean variables and variances of perturbations, by which the true variance $\sigma_{w}^{2}$ tends to be overestimated. For example, upon variation of pitch angle, part of the mean along‐track wind $\bar{u}$ is sampled and this effect appears as an additional contribution $\sigma_{\theta }^{2}{\bar{u}}^{2}$ to the true variance $\sigma_{w}^{2}$. Terms 8–12 of Eq. (A7) consist of products of mean variables and covariances of the perturbation variables, which can assume either sign.

Since the horizontal along‐track and cross‐track components of the wind *u* and *v* in each radar range gate are not known, it is not possible to evaluate these terms exactly. Instead, we must seek an upper bound for the uncertainty in the estimation of $\sigma_{w}^{2}$ that they cause. We define the upper bounds of terms 2–7 and terms 8–12 of Eq. (A7) as, respectively,

(A8) $$\left.\begin{array}{cc} \sigma_{\text{HC},A}^{2}= & \sigma_{\theta }^{2}{\bar{u}}^{2}+{\bar{\theta }}^{2}{\sigma_{u}}^{2}+\sigma_{\theta }^{2}{\sigma_{u}}^{2}\\ +\sigma_{\varphi }^{2}{\bar{v}}^{2}+{\bar{\varphi }}^{2}{\sigma_{v}}^{2}+\sigma_{\varphi }^{2}{\sigma_{v}}^{2},\\ \sigma_{\text{HC},B}^{2}= & \left|2\bar{\theta }\bar{u^{{}^{\prime }}w^{{}^{\prime }}}|+|2\bar{\varphi }\bar{v^{{}^{\prime }}w^{{}^{\prime }}}|+|2\bar{\theta^{{}^{\prime }}\varphi^{{}^{\prime }}}\bar{u^{{}^{\prime }}v^{{}^{\prime }}}\right|\\ +|2\bar{\theta^{{}^{\prime }}\varphi^{{}^{\prime }}}\bar{u}\bar{v}|+|2\bar{\theta }\bar{\varphi }\bar{u^{{}^{\prime }}v^{{}^{\prime }}}|. \end{array}\;\right\}$$

All quantities printed in boldface are unknowns and we approximate them by making the following assumptions:

- $\bar{u}\leq {\bar{u}}_{\text{max}}≃30ms^{-1}$. The minimum and maximum along‐track wind components are −5 m s^−1^ (in regions of reversed flow near the ground) and 25 m s^−1^, respectively;
- $\bar{v}\leq {\bar{v}}_{\text{max}}≃10ms^{-1}$, which corresponds to the maximum cross‐track wind component;
- $\sigma_{u}^{2}≃\sigma_{v}^{2}≃\sigma_{v_{r}}^{2}$;
- $|\bar{u^{{}^{\prime }}v^{{}^{\prime }}}|≃2\sigma_{r_{\mathrm{uv}}}\cdot \sigma_{v_{r}}^{2}$, where $2\sigma_{r_{\mathrm{uv}}}$ is two times the standard deviation of correlation coefficients $r_{\mathrm{uv}}=\bar{u^{{}^{\prime }}v^{{}^{\prime }}}/(\sigma_{u}\sigma_{v})$ obtained from the *in situ* data. Accordingly, $|\bar{u^{{}^{\prime }}w^{{}^{\prime }}}|≃2\sigma_{r_{\mathrm{uw}}}\sigma_{v_{r}}^{2}$ and $|\bar{v^{{}^{\prime }}w^{{}^{\prime }}}|≃2\sigma_{r_{\mathrm{vw}}}\sigma_{v_{r}}^{2}$. We determined $\sigma_{r_{\mathrm{uv}}}$, $\sigma_{r_{\mathrm{uw}}}$, and $\sigma_{r_{\mathrm{vw}}}$ as 0.305, 0.294, and 0.291, respectively, considering *in situ* measurements from all flights on 26 January and on 5 February.

The approximated upper bounds $\sigma_{\text{HC},A}^{2}$ and $\sigma_{\text{HC},B}^{2}$ to the horizontal wind contamination are now given by

(A9) $$\left.\begin{array}{cc} \sigma_{\text{HC},A}^{2}\;= & \sigma_{\theta }^{2}{{\bar{u}}_{\text{max}}}^{2}+{\bar{\theta }}^{2}{\sigma_{v_{r}}}^{2}+\sigma_{\theta }^{2}{\sigma_{v_{r}}}^{2}\\ +\sigma_{\varphi }^{2}{{\bar{v}}_{\text{max}}}^{2}+{\bar{\varphi }}^{2}{\sigma_{v_{r}}}^{2}+\sigma_{\varphi }^{2}{\sigma_{v_{r}}}^{2},\\ \sigma_{\text{HC},B}^{2}\;= & 2|\bar{\theta }|\cdot 2\sigma_{r_{\mathrm{uw}}}\sigma_{v_{r}}^{2}+2|\bar{\varphi }|\cdot 2\sigma_{r_{\mathrm{vw}}}\sigma_{v_{r}}^{2}\\ \;+2|\bar{\theta^{{}^{\prime }}\varphi^{{}^{\prime }}}|\;\cdot \;2\sigma_{r_{\mathrm{uv}}}\sigma_{v_{r}}^{2}\;\;\;+\;2|\bar{\theta^{{}^{\prime }}\varphi^{{}^{\prime }}}|\;\cdot \;{\bar{u}}_{\text{max}}{\bar{v}}_{\text{max}}\\ \;+2|\bar{\theta }\bar{\varphi }|\cdot 2\sigma_{r_{\mathrm{uv}}}\sigma_{v_{r}}^{2}, \end{array}\;\right\}$$

and the upper and lower uncertainty bounds of $\sigma_{w}^{2}$ due to the horizontal wind contamination can be written, respectively, as

(A10) $$\sigma_{w}^{2}=\sigma_{v_{r}}^{2}+\left\{\begin{array}{ll} +\sigma_{\text{HC},B}^{2}\;,\\ -\sigma_{\text{HC},A}^{2} & -\sigma_{\text{HC},B}^{2}. \end{array}\right.$$

It is apparent from Eq. (A9) that the (upper‐bounded) uncertainty in the estimation of $\sigma_{w}^{2}$ due to the horizontal wind contamination of $\sigma_{v_{r}}^{2}$ is highly variable and mainly depends on aircraft pitch *θ* and roll *φ* and on $\sigma_{v_{r}}^{2}$ itself. The main contribution to the uncertainty can be traced back to the terms $\sigma_{\theta }^{2}{\bar{u}}_{\text{max}}^{2}$, $2|\bar{\theta }|\;\cdot \;2\sigma_{r_{\mathrm{uw}}}\sigma_{v_{r}}^{2}$ and $2|\bar{\varphi }|\;\cdot \;2\sigma_{r_{\mathrm{vw}}}\sigma_{v_{r}}^{2}$ in Eq. (A9), making up for roughly 90% of the total. Figure A1 shows two examples of the relative uncertainty of $\sigma_{w}^{2}$ due to the horizontal wind contamination, defined as $(\sigma_{\text{HC},A}^{2}+\sigma_{\text{HC},B}^{2})/\sigma_{v_{r}}^{2}$. Regions in the lower‐level flow with increased relative uncertainty are associated with patches of moderate turbulence at flight level. The relative uncertainty can be substantial if the sampled atmospheric turbulence is low, but it remains below 20% in the most turbulent regions of the flow.

## Appendix B 1

### Uncertainty in the estimation of energy dissipation rate from radar data

In section 3.3, we link the primary uncertainty in the estimation of EDR from aircraft *in situ* and Doppler radar measurements to the scatter of individual EDR_*i*_ estimates around the mean $\bar{\text{EDR}}$. The uncertainties of individual EDR_*i*_ estimates and the mean $\bar{\text{EDR}}$ are found to be ±29 and ±16% respectively.

For EDR estimates from radar data, two additional possible sources of error have to be taken into account: the contamination of $\sigma_{v_{r}}^{2}$ by the horizontal wind and the uncertainty in the along‐track wind in a given radar range gate. The first term contributes less than 0.5% uncertainty. In fact, with the assumption of locally isotropic turbulence, the spectral power densities *S*_*w*_(*k*) and $S_{v_{r}}(k)$ differ only by a factor $S_{w}(k)/S_{v_{r}}(k)=4/3{\left\{1+1/3\overset{2}{\cos}(\theta )\right\}}^{-1}$, which never exceeds 1.0038 for a maximum pitch angle of $\theta_{\text{max}}≃6^{\circ }$≃0.11 rad. We thus neglect this term.

The second uncertainty results from the error made in the estimation of EDR from the vertical Doppler velocity,

(B1) $${\text{EDR}}_{w}={ɛ}_{w}^{1/3}={\left[\frac{2\pi }{\text{TAS}}{\left(\frac{\bar{S_{v_{r}}(f)f^{5/3}}}{\alpha_{w}}\right)}^{3/2}\right]}^{1/3},$$

due to the conversion of WCR time series to spatial series using the aircraft true airspeed TAS at flight level,

(B2) $$\text{TAS}=\text{GSPD}-u_{\text{fl}}.$$

GSPD is the aircraft speed relative to the ground and *u*_fl_ is the along‐track wind component at flight level. Far from flight level, *u* deviates up to 30 m s^−1^ from *u*_fl_, giving rise to a maximum relative error in EDR_*w*_ of

(B3) $$\begin{array}{lll} \frac{{\text{EDR}}_{w}}{\text{EDR}}-1= & {\left(\frac{\text{TAS}\pm 30ms^{-1}}{\text{TAS}}\right)}^{1/3}-1\; & \\ ≃ & \left\{\begin{array}{ll} +9\% & \text{for a tailwind,}\\ -11\% & \text{for a headwind,} \end{array}\right.\; & \; \end{array}$$

for an average TAS of 100 m s^−1^. The total uncertainty in the estimation of EDR_*w*_ from radar data is thus given by

(B4) $$\begin{array}{lll} \text{EDR}= & ({{\text{EDR}}_{w}}_{-11\%}^{+9\%})\pm 29\%\; & \;\\ = & \left\{\begin{array}{ll} {{\text{EDR}}_{w}}_{-23\%}^{+41\%} & \text{for a tailwind,}\\ {{\text{EDR}}_{w}}_{-37\%}^{+15\%} & \text{for a headwind.} \end{array}\right.\; & \end{array}$$
